# MGAT4A/Galectin9‐Driven *N*‐Glycosylation Aberration as a Promoting Mechanism for Poor Prognosis of Endometrial Cancer with *TP53* Mutation

**DOI:** 10.1002/advs.202409764

**Published:** 2024-11-11

**Authors:** Zhen Zhu, Jingya Sun, Weiqing Xu, Qinghe Zeng, Hanyi Feng, Lijuan Zang, Yinyan He, Xiao He, Na Sheng, Xuelian Ren, Guobin Liu, He Huang, Ruimin Huang, Jun Yan

**Affiliations:** ^1^ Center for Medical Research and Innovation Shanghai Pudong Hospital Fudan University Pudong Medical Center; Laboratory Animal Center Fudan University Shanghai 200032 China; ^2^ Model Animal Research Center of Nanjing University Nanjing 210061 China; ^3^ Center for Drug Safety Evaluation and Research Shanghai Institute of Materia Medica Chinese Academy of Sciences Shanghai 201203 China; ^4^ Department of Pathology Shanghai General Hospital Shanghai Jiao Tong University School of Medicine Shanghai 200080 China; ^5^ School of Chinese Materia Medica Nanjing University of Chinese Medicine Nanjing 210023 China; ^6^ Department of Obstetrics and Gynecology Shanghai General Hospital Shanghai Jiao Tong University School of Medicine Shanghai 200080 China; ^7^ Department of Obstetrics and Gynecology Shanghai Tenth People's Hospital Tongji University School of Medicine Shanghai 200072 China; ^8^ State Key Laboratory of Chemical Biology Shanghai Institute of Materia Medica Chinese Academy of Sciences Shanghai 201203 China; ^9^ University of Chinese Academy of Sciences Beijing 100049 China; ^10^ School of Pharmaceutical Science and Technology Hangzhou Institute for Advanced Study University of Chinese Academy of Sciences Hangzhou 310024 China

**Keywords:** endometrial cancer, glucose metabolism, MGAT4A, *N*‐glycosylation, *TP53* mutation

## Abstract

Emerging evidence recognizes aberrant glycosylation as the malignant characteristics of cancer cells, but little is known about glycogenes’ roles in endometrial carcinoma (EC), especially the most aggressive subtype carrying *TP53* mutations. Using unsupervised hierarchical clustering, an 11‐glycogene cluster is identified to distinguish an EC subtype associated with frequent *TP53* mutation and worse prognosis. Among them, MGAT4A (alpha‐1,3‐mannosyl‐glycoprotein 4‐β‐*N*‐acetylglucosaminyltransferase A) emerges as the most consistently overexpressed glycogene, contributing to EC aggressiveness. In the presence of galectin‐9, MGAT4A increases EC cell proliferation and invasion via promoting glucose metabolism. *N‐*glycoproteomics further revealed GLUT1, a glucose transporter, as a glycoprotein modified by MGAT4A. Binding of galectin‐9 to the MGAT4A‐branched *N‐*glycan on GLUT1 enhances its cell membrane distribution, leading to glucose uptake increase. In addition, oncogenic mutations of *TP53* gene in EC cells upregulate MGAT4A expression by disrupting the regulatory oversight exerted by wild‐type p53 on tumor‐suppressive miRNAs, including miR‐34a and miR‐449a/b. The findings highlight a new molecular mechanism involving MGAT4A‐regulated *N‐*glycosylation on the key regulator of glucose metabolism in p53 mutants‐driven EC aggressiveness, which may provide a strategic avenue to combat advanced EC.

## Graphical abstract

Aberrant glycosylation is a hallmark of cancer cells, yet the understanding of glycogenes' contribution to endometrial carcinoma (EC) remains limited, particularly the most aggressive subtype carrying *TP53* mutations. This study reveals p53‐mutants promote EC aggressiveness via miRNAs, *N*‐glycosylation on GLUT1 with the interaction of galectin‐9 to upregulate glucose metabolism, centered on MGAT4A.

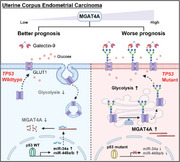

## 1 Introduction

Endometrial cancer (EC) is the sixth most common malignancy in women globally, with higher incidence rates in developed countries than developing countries.^[^ ^1^ ^]^ Unfortunately, its overall incidence worldwide has increased by 132% between 1990 and 2019 and a rise of EC incidence is projected to become the third leading cancer type among women in the United States by 2030.^[^ ^2^, ^3^, ^4^ ^]^ Though women with early stage EC have better prognosis, the clinical outcomes for primary advanced or recurrent EC patients are poor, with median overall survival of less than 3 years.^[^ ^5^, ^6^ ^]^ Therefore, it is needed to molecularly stratify EC patients with different clinical outcomes and to identify critical drivers of EC aggressiveness for potential drug targets.

Usually ECs are divided into two pathogenetic subgroups: Type I refers to low‐grade endometrioid ECs with well‐ or moderate‐differentiated status, whereas Type II is enriched with high‐grade endometrioid and serous ECs with poorer differentiated status. Recent molecular stratification categorizes ECs into four genomic subtypes: POLE ultramutated, hypermutated microsatellite instability (MSI), copy‐number low, and copy‐number high, which show different prognoses and clinical implications.^[^ ^7^, ^8^ ^]^ Among them, the copy‐number high group displays worse progression‐free survival than other groups and contains high *TP53* mutation rate (90%) and few *PTEN* mutation rate (11%), while few *TP53* mutation and high *PTEN* mutation rates are detected in other three groups.^[^ ^7^, ^9^ ^]^ In addition, the treatment of immune checkpoint inhibitors benefits EC patients with MSI subtype, while trastuzumab is suggested for HER2 positive copy‐number high subtype in NCCN compendium.^[^ ^10^ ^]^ Overall, such molecular classification shows prognosis prediction and strong therapeutic implications.

As a common phenomenon during carcinogenesis, aberrant glycosylation affects protein folding, distribution, and their functions on cell proliferation, survival, metastasis, and immune evasion.^[^ ^11^, ^12^ ^]^ A further featurization of the glycosylation abnormity of proliferating and invading cancer cells has led to the discovery of novel cancer antigens and an improved understanding of the efficacy of cancer therapies.^[^ ^13^, ^14^, ^15^ ^]^ The changes of the *N‐*linked glycoprotein synthesis and glycosylation profiles are frequently detected in normal endometrial cells during the menstrual cycle, and in various disease statuses, such as endometriosis and EC development.^[^ ^16^, ^17^, ^18^, ^19^ ^]^ Glycosylation also significantly impacts the function and efficacy of immunomodulatory molecules such as PD‐L1, CTLA‐4, CD28, CD40/CD40L, TIM‐3, Siglecs, and Selectins by modulating stability, ligand interaction, and cell signaling, affecting immune responses, and immunotherapy.^[^ ^20^, ^21^, ^22^ ^]^ Therefore, it is intriguing to classify EC subtypes in the perspective of glycogenes (the glycosylation‐related genes, including glycosyltransferases, glycosidases, and nucleotide sugar synthesis and transporter genes). Previously, Noda et al. and our groups have showed that glycogene‐based classification successfully stratified cancer into different prognostic subtypes, tightly associated with specific subtypes of colon cancer and bladder cancer, respectively.^[^ ^23^, ^24^ ^]^ Hence, we hypothesize that EC could also be explored to distinguish the subtype with poor prognosis and to promisingly provide therapeutic implications, based on glycogene expression profiling.

Altered glucose metabolism is a well‐known characteristic of cancer, contributing to various hallmarks of malignancy. Tumor cells exploit aerobic glycolysis to fuel cell growth, which produces ATP with lower efficiency than oxidative phosphorylation. They generally upregulate glucose transporters, particularly GLUT1, to substantially increase glucose uptake.^[^ ^25^ ^]^ Interestingly, a strong link between diet‐related glucose elevation and high risk for EC was indicated by a recent epidemiologic study,^[^ ^26^, ^27^ ^]^ while high glucose culture condition led to abnormal glucose metabolism, increased EC cell proliferation, and invasion.^[^ ^28^ ^]^ Moreover, elevated expression of GLUT1 was considered as an essential early step in the progression and drug resistance of EC.^[^ ^29^ ^]^ However, the regulation of GLUT1 in EC cells and the role of glycosylation in promoting glucose metabolism reprogramming and EC aggressiveness remain to be fully understood.

Herein, we established a novel glycogene‐based molecular classification for EC patients. A glycosyltransferase, MGAT4A, which initiates the first step of *N‐*glycosylation β1,4‐GlcNAc branches, showed a significant upregulation in the more aggressive population of EC patients. How MGAT4A promoted EC progression and how MGAT4A mRNA was upregulated in EC cells were further investigated. A regulatory axis involving p53/miRNAs/MGAT4A‐galectin‐9 (GAL9) on GLUT1‐dependent glucose metabolism in EC patients with *TP53* mutations, which have worse prognosis, was demonstrated.

## 2 Results

### 2.1 Glycogene‐Defined Subtypes were Associated with Clinical, Genetic and Molecular Features of EC Patients

**Figure 1:**
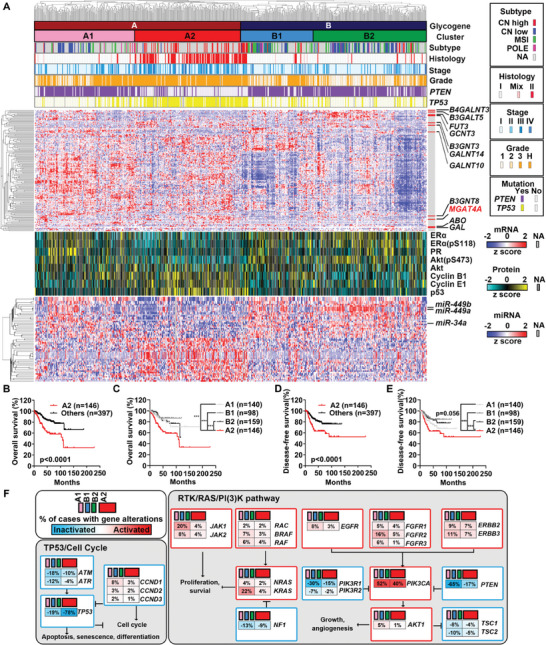
Glycogene‐defined subtypes were associated with clinical, genetic, and molecular features of ECs. A) Classification of EC patients from TCGA‐UCEC cohort into four clusters (A1, A2, B1, and B2) based on glycogene expression. Clustering for TCGA subtype, histology, stage, grade, gene mutations in *PTEN* and *TP53*, glycogene expression, normalized protein expression, and miRNA expression were also respectively shown. B–E) Kaplan–Meier plots of EC patients’ outcomes in TCGA‐UCEC cohort, comparing those in Cluster A2 with other three clusters (A1, B1, and B2) (B, overall survival; with A1, B1, and B2 together; C, overall survival; with A1, B1, and B2 separately; D, disease‐free survival; with A1, B1, and B2 together; E, disease‐free survival; with A1, B1, and B2 separately). F) Comparison genetic mutations of TP53/cell cycle and RTK/RAS/PI3K pathway between Cluster A2 and other three clusters.

To elucidate the role of glycogenes in EC and their association with clinical outcomes, we conducted a comprehensive analysis of EC patients from the TCGA‐UCEC cohort (*n* = 543; Table S1, Supporting Information). The results showed that EC patients could be divided into four clusters (A1, A2, B1, and B2), each defined by unique pathological and clinical characteristics (**Figure** **1**A). Cluster A2 was significantly correlated with advanced‐stage, high‐grade EC and predominantly exhibited Type II histology (Figure 1A). This cluster was associated with poorer outcomes compared to other clusters (including A1, B1, and B2, in combination or separately; Figure 1B–E), demonstrating the capability of glycogene‐based classification in identifying EC patients with more aggressive and advanced disease profiles. Further analysis revealed the distinct genetic characteristics within Cluster A2 compared to other clusters. The gene mutation profiles were markedly different, with a significant enrichment of “TP53/cell cycle pathway” in Cluster A2, differing significantly from other clusters in which “RTK/RAS/PI3K pathway” mutations were more prevalent (mutation rate >10%, the oncogenic mutations in pathway were showed in Figure 1F). This divergence was substantiated by proteomic data from the TCGA‐UCEC cohort, which also indicated variations in ER and PR activation between Cluster A2 and other clusters (Figure 1A).

Those clinical, genetic, and molecular differences indicated that EC patients in Cluster A2 followed a divergent oncogenic pathway with the differential expression of glycogenes.

### 2.2 Associations Between MGAT4A and Pathological/Molecular Features of EC Patients

**Figure 2:**
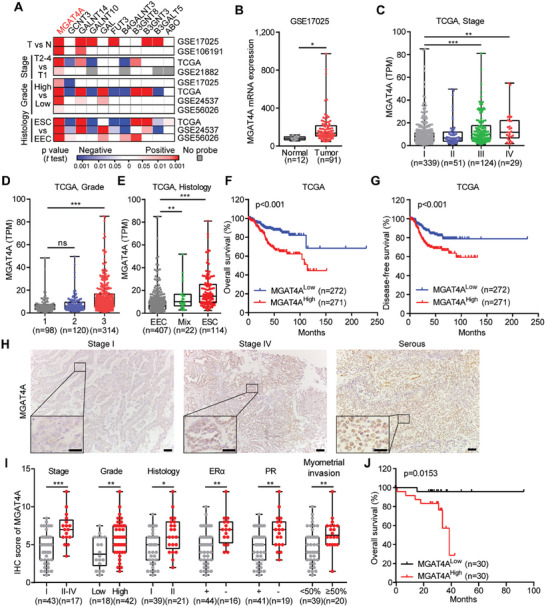
The association of MGAT4A expression with clinical features in ECs. A) Heatmap showed the association of differentially expressed glycogenes in Cluster A2 with clinical relevance across multiple EC cohorts. Colored‐blocks in the heatmap represented the glycogenes with upregulation (in red) or downregulation (in blue), respectively. B) MGAT4A mRNA levels in normal and EC tissues from GSE17025 cohort. C–E) The association of MGAT4A mRNA level with tumor stage (I‐IV, C), grade (1‐3, D), and histology (endometrial endometrioid carcinoma (EEC), endometrial serous carcinoma (ESC), and Mix, E) in TCGA‐UCEC cohort. F,G) Kaplan–Meier plots depicting the overall survival (F) and disease‐free survival (G) of EC patients in TCGA‐UCEC cohort, stratified by MGAT4A mRNA level. H) IHC staining for MGAT4A protein in EC specimens from our own cohort (*n* = 60). Images from three representative EC cases including stage I, stage IV, and serous histology were included. Scale bar, 200 µm; inset, 20 µm. I) The association of MGAT4A IHC score with tumor stage (I or II‐IV), grade (low or high), histology (Type I or II), ERα status (positive or negative), PR status (positive or negative), and myometrial invasion status (<50% or ≥50%). J) Kaplan–Meier plots of overall survival of EC patients from our own cohort, stratified by MGAT4A IHC score. *p*‐values were calculated with unpaired Student's *t*‐test (B and I) and one‐way ANOVA (C–E). ^*^ *p* < 0.05; ^**^ *p* < 0.01; ^***^ *p* < 0.001; ns, *p* ≥ 0.05.

Since Cluster A2 by this glycogene‐based classification was notably correlated with poor clinical outcomes, our focus shifted to pinpoint the key glycogenes integrally linked to this high‐risk cluster and its related clinical manifestations. Subsequent KEGG pathway analysis revealed that “*N‐*glycan biosynthesis” was the most significantly enriched glycosylation‐relating pathway in Cluster A2 (Figure S1A, Supporting Information). To find out the key glycogenes, we screened the ones differentially expressed in Cluster A2 comparing with other clusters, revealing 11 glycogenes with notable differential expression (*p* < 0.05, false discovery rate (FDR) < 0.05; **Figure** **2**A; Figure S1B; Table S2, Supporting Information). Among these, MGAT4A (alpha‐1,3‐mannosyl‐glycoprotein 4‐β‐*N*‐acetylglucosaminyltransferase A) showed a consistent upregulation in patients with high stage, high grade, and aggressive histological variants across multiple cohorts (Figure 2A–E; Figure S2A–F, Supporting Information). Notably, MGAT4A overexpression was associated with higher mortality and was predictive of poorer prognosis in TCGA‐UCEC and GSE21882 cohorts (Figures 2F,G; S2G, Supporting Information).

MGAT4A, a type II transmembrane protein, facilitates the addition of uridine diphosphate *N‐*acetylglucosamine (UDP‐GlcNAc) to the C‐4 position of core α1‐3 Man residues. This process leads to the formation of tri‐ and tetra‐antennary *N‐*glycans on both membrane‐bound and secreted proteins within the Golgi apparatus, thereby contributing to LacNAc modification (Figure S2H, Supporting Information).^[^ ^30^ ^]^ As the initiated enzymes catalyzing β1,4‐GlcNAc branches in *N‐*glycan, MGAT4A was predominantly expressed in tumor cells, while MGAT4B was found in tumor‐infiltrated stromal cells as per the Human Protein Atlas endometrial cancer database (Figure S2I, Supporting Information). MGAT4B expression did not show significant differences across different stages nor in clinical outcomes in the TCGA‐UCEC cohort (Figure S2J,K, Supporting Information), indicating a unique role of MGAT4A in EC development. Besides, the elevated levels of MGAT4A protein and β1,4‐GlcNAc‐branched *N*‐glycan detected by Datura Stramonium Lectin (DSL) binding were observed in EC tissues, compared to their adjacent noncancerous endometrial tissues (Figure S2L, Supporting Information), indicating that these alterations might be specific to EC. Furthermore, in our own cohort containing 60 EC specimens, high MGAT4A protein expression by immunohistochemistry (IHC) assay was significantly associated with advanced tumor stage, grade, histology status, ER/PR‐negative status, as well as myometrial invasion (Figure 2H,I; Table S3, Supporting Information). Using a combination of MGAT4A protein levels (by IHC scores) and above clinicopathological parameters, these 60 EC tissues were clustered, and patients in Cluster A exhibited similar characteristics as the ones in Cluster A2 from the TCGA cohorts (Figure S1B,C, Supporting Information). Higher MGAT4A protein level was also indicated worse prognosis in EC patients (*p* = 0.0153; Figure 2J).

Taken together, our findings confirmed that MGAT4A, a glycosyltransferase catalyzes the GlcNAc transfer, was upregulated in the high‐risk EC patients and exhibited a significant association with clinicopathological and molecular features of aggressive EC.

### 2.3 MGAT4A Mediated β1,4‐GlcNAc Modification and Promoted EC Cell Proliferation and Invasion with GAL9

**Figure 3:**
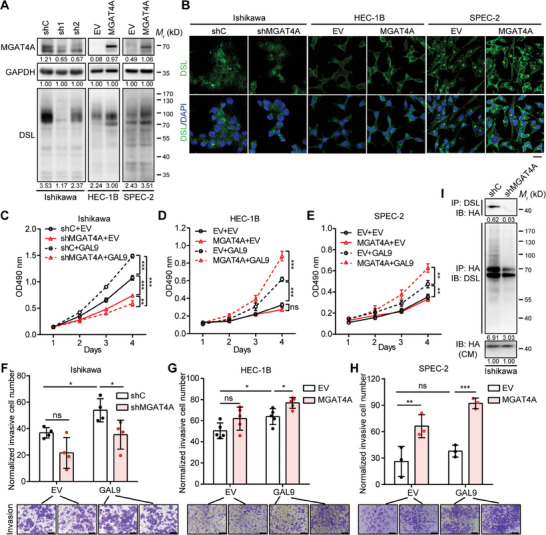
MGAT4A mediated β1,4‐GlcNAc modification and promoted EC cell proliferation and invasion with GAL9. A) Effects of MGAT4A protein expression on the levels of (β‐1,4) linked *N*‐acetylglucosamine oligomers on glycoproteins by DSL binding in Ishikawa cells with MGAT4A knockdown (sh1 and sh2; shC, the negative control), and in HEC‐1B and SPEC‐2 cells with ectopic MGAT4A expression (EV, empty vector control), respectively. B) IF staining by DSL for the levels of β1,4‐GlcNAc branch modification in Ishikawa cells with MGAT4A knockdown, as well as in HEC‐1B and SPEC‐2 cells with ectopic MGAT4A expression. Scale bar, 20 µm. C–E) The proliferation of Ishikawa (C), HEC‐1B (D) and SPEC‐2 cells (E) with the knockdown/overexpression of MGAT4A and GAL9 by MTT assay. F–H) The invasion of Ishikawa (F), HEC‐1B (G) and SPEC‐2 cells (H) with the knockdown/overexpression of MGAT4A and GAL9 by Transwell assay. Scale bar, 100 µm. I) GAL9 binding to glycoproteins modified by MGAT4A in Ishikawa cells with MGAT4A knockdown and HA‐tagged GAL9 overexpression. Glycoproteins with β1,4‐GlcNAc modification were IP by DSL and the ectopic expressed HA‐tagged GAL9 protein was detected by HA‐tag antibody, and vice versa. The GAL9 level in conditional medium (CM) was used as the loading control. Data were presented as mean ± SD from three independent experiments. *p*‐values were calculated with two‐way ANOVA (C–H). ^*^ *p* < 0.05; ^**^ *p* < 0.01; ^***^ *p < *0.001; ns, *p* ≥ 0.05.

Given the association of MGAT4A with aggressive EC, we investigated its role in cancer progression. MGAT4A protein level was found to be higher in Ishikawa cells, while lower in HEC‐1B, AN3‐CA, and SPEC‐2 cells (Figure S3A, Supporting Information). Given the comparable doubling time of Ishikawa, HEC‐1B and SPEC‐2 cells, we stably knocked down MGAT4A in Ishikawa cells and ectopically expressed MGAT4A in HEC‐1B and SPEC‐2 cells, respectively (**Figure** **3**A). MGAT4A knockdown resulted in a marked decrease in β1,4‐GlcNAc branch modification in Ishikawa cells, as indicated by DSL binding; while MGAT4A overexpression in SPEC‐2 and HEC‐1B cells increased the levels of (β‐1,4) linked *N*‐acetylglucosamine oligomers (Figure 3A). Immunofluorescence (IF) staining further confirmed the DSL‐binding alterations in these EC cells in situ (Figure 3B). Notably, MGAT4A knockdown dramatically inhibited Ishikawa cell proliferation (*p* < 0.001; Figure 3C); but its overexpression did not significantly affect HEC‐1B and SPEC‐2 cell proliferation (Figure 3D,E). These data suggested that there might be additional factors to promote EC cell proliferation in combination with MGAT4A overexpression.

Branching of *N‐*glycosylation by the MGATs, known to increase the affinity for galectins, is implicated in carcinogenesis.^[^ ^31^ ^]^ Galectin recognizes LacNAc‐modified glycoproteins, contributing to the stability and/or function of membrane glycoproteins (Figure S2H, Supporting Information).^[^ ^32^, ^33^, ^34^, ^35^, ^36^ ^]^ Since MGAT4A initiates β1,4‐LacNAc branch modification of membrane glycoprotein, we hypothesized that galectins interact with MGAT4A‐modified glycoproteins and mediate their specific cellular function in EC. Screening the galectins in public EC cohorts thus revealed the increased mRNA expression of *LGALS7B* and *LGALS9* genes in EC samples from TCGA cohort (*p* < 0.001), and only *LGALS9* exhibited higher expression in EC samples from GSE17025 cohort (*p* < 0.01; Figure S3B, Supporting Information). A series of galectins, including GAL‐1, 2, 3, 7B, 8, and 9, were ectopically expressed in Ishikawa cells respectively, confirmed by their presence in the whole cell lysate (WCL) and conditional medium (CM) (Figure S3C, Supporting Information). DSL immunoprecipitation (IP) indicated the interactions between glycoproteins with β1,4‐GlcNAc and these galectins (Figure S3C, Supporting Information). We also observed that CM from Ishikawa cells with the overexpressed GAL8 and GAL9 consistently promoted both Ishikawa and HEC‐1B cell proliferation (Figure S3D,E, Supporting Information), as well as CM from Ishikawa cells with the overexpressed GAL2, GAL3, GAL7B, and GAL9 enhanced Ishikawa cell invasiveness, respectively (Figure S3F, Supporting Information). Thus, GAL9 was chosen for further confirmation. To exclude the possible interference of other components in CM from GAL9‐overexpressing Ishikawa cells, the recombinant human galectin‐9 protein (rhGAL9) was applied. As shown in Figure S3G,H (Supporting Information), rhGAL9 (2 µg mL^−1^) promoted both the proliferation and invasiveness of Ishikawa cells (*p* < 0.05), whereas it did not have such promoting effects on Ishikawa cells with MGAT4A knockdown. GAL9 as a major contributor to the cell proliferation and invasion‐promotion dependent on MGAT4A was indicated.

To further confirm the effects of GAL9 overexpression on EC cells, we introduced exogenous HA‐tagged GAL9 in Ishikawa, HEC‐1B and SPEC‐2 cells (Figure S3I, Supporting Information). GAL9 overexpression alone boosted proliferation of Ishikawa (*p* < 0.001; Figure 3C), HEC‐1B (*p *< 0.001; Figure 3D) and SPEC‐2 cells (*p* < 0.01; Figure 3E); however, it could only significantly increase the invasion of Ishikawa and HEC‐1B cells (*p* < 0.05; Figure 3F–H). Moreover, in GAL9‐overexpressing Ishikawa cells, MGAT4A knockdown resulted in significantly reduced proliferation and invasiveness (*p* < 0.05; Figure 3C,F); similarly, in GAL9‐overexpressing HEC‐1B and SPEC‐2 cells, the ectopic expression of MGAT4A led to significantly increase proliferation and invasiveness (*p* < 0.05; Figure 3D,E,G,H). Both DSL IP and HA‐tag IP showed that MGAT4A knockdown in HA‐tagged GAL9 overexpressing Ishikawa cells led to less binding of HA‐tagged GAL9 to glycoproteins modified by MGAT4A evidenced by DSL binding (Figure 3I).

Collectively, these results showed that MGAT4A overexpression promoted EC cell proliferation and invasion, in presence of GAL9, via their cooperative regulation on *N‐*glycan branch modification of glycoproteins.

### 2.4 MGAT4A/GAL9‐Driven *N‐*glycosylation Increased GLUT1 Membrane Localization

**Figure 4:**
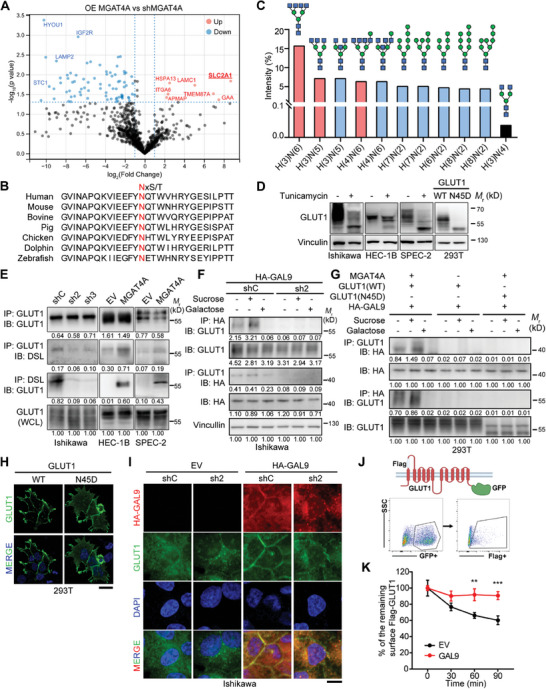
MGAT4A/GAL9‐driven *N‐*glycosylation increased GLUT1 membrane localization. A) *N‐*glycoproteomics analysis of Ishikawa cells with MGAT4A overexpression (OE MGAT4A) or knockdown (shMGAT4A). Proteins with different *N‐*glycan modifications were exhibited as volcano plots. Upregulation, red dot; downregulation, blue dot. SLC2A1, official name of GLUT1. B) The amino acid sequences of GLUT1 protein containing the predicted glycosylation site (Asn45, in red) across different species. C) The predicted glycan structures on GLUT1 glycoprotein at Asn45 in 293T cells co‐transfected with Flag‐GLUT1, MGAT4A and GAL9, characterized by mass spectrometry based glycoproteomics. D) Western blot analyses of GLUT1 in Ishikawa, HEC‐1B and SPEC‐2 cells treated with 10 µg mL^−1^ tunicamycin, an inhibitor of *N‐*linked glycosylation. The WT and N45D mutated (Asn45 mutated to Asp45, interfering the *N‐*glycosylation modification) GLUT1 proteins were expressed in 293T cells, respectively. E) Co‐IP assays for assessment of β1,4‐GlcNAc modification level, recognized by DSL, on GLUT1 protein mediated by MGAT4A in Ishikawa cells, HEC‐1B and SPEC‐2 cells. F,G) Co‐IP assays for binding of HA‐tagged GAL9 to GLUT1 glycoprotein mediating by MGAT4A in Ishikawa (F) and 293T cells (G). Cells were treated with galactose (10 mmol L^−1^, to block the GAL9 binding to LacNAc in carbohydrate chain) or sucrose (10 mmol L^−1^, a negative control). H) IF staining for GLUT1 (green) in 293T cells transfected GLUT1‐WT or ‐N45D mutant. DAPI (blue) was used for nuclei staining. Scale bar, 20 µm. I) IF co‐staining for HA‐tagged GAL9 (red) and GLUT1 (green) in Ishikawa cells with MGAT4A knockdown and GAL9 ectopic expression. DAPI (blue) was used for nuclei staining. Scale bar, 20 µm. J) Schematic diagram of the membrane‐bound GLUT1 reporter, *N‐*terminal tagged Flag‐GLUT1 protein fused with GFP at its C‐terminal. K) Flow cytometry analysis of the membrane‐bound GLUT1 reporter (J) in 293T cells expressing GAL9 or an empty vector (EV). Cells were pretreated with Flag M2 antibody, followed by a specified period (0‐90 min) for Flag‐tagged GLUT1 internalization. The membrane‐bound GLUT1 was defined as the Flag^+^GFP^+^ population by flow cytometry. *p*‐values were calculated with unpaired Student's *t*‐test (*K*). ^**^ *p* < 0.01; ^***^ *p* < 0.001.

We further performed the *N‐*glycoproteomics analysis to identify MGAT4A's potential substrate proteins in Ishikawa cells. The MGAT4A‐overexpressed cells were compared with the MGAT4A‐knockdown cells for *N‐*glycosylation alterations. Seven genes exhibited *N‐*glycosylation increase in response to MGAT4A overexpression (fold change ≥ 1.5, *p* < 0.05; **Figure** **4**A). Remarkably, Glucose Transporter member 1 (GLUT1), which was encoded by *SLC1A1*, was most significantly modified by glycosylation. To delineate the glycan structures presented on GLUT1, a site‐specific *N‐*glycosylation analysis was carried out. 293T cells were co‐transfected with Flag‐GLUT1, MGAT4A, and GAL9, followed by IP assay with the Flag antibody for further *N‐*glycosylation analysis. A total of 84 glycan variants at the GLUT1 N45 site were discerned, which was consistent with that Asparagine 45 was species‐conservative with a potential *N‐*glycosylation site (NxS/T) (Figure 4B). Ten most prevalent structures were depicted in Figure 4C. The direct or subsequent reaction products catalyzed by MGAT4A, including H(3)N(6), H(3)N(5), and H(4)N(6), were cumulated representing 29.08% of the glycan profile (indicated in red; Figure 4C); whereas H(3)N(4), the substrate of MGAT4A, only remained 0.04% (indicated in black; Figure 4C). These results demonstrated that MGAT4A regulated the LacNAc modification of GLUT1 at N45 residue.

Glucose transporters are membrane proteins that facilitate glucose across the plasma membrane, which have been reported with *N‐*glycan modification.^[^ ^37^ ^]^ Disruption of MGAT4A in murine models has been linked to hyperglycemia and anomalous activities of GLUT2 in pancreatic cells.^[^ ^38^ ^]^ Thus, we employed tunicamycin, an inhibitor of *N‐*linked glycosylation,^[^ ^39^ ^]^ to ascertain the *N‐*glycosylation status of GLUT1 in EC cells. A remarkable shift of endogenous GLUT1 band was observed in Ishikawa, HEC‐1B and SPEC‐2 cells with tunicamycin treatment, as well as in 293T cells with GLUT1 mutant protein (N45D) at *N‐*glycan site, compared with wild‐type GLUT1 (GLUT1‐WT) protein (Figure 4D). To investigate whether *N‐*glycosylation on GLUT1 was mediated by MGAT4A, the IP assay for GLUT1 was carried out. The DSL signal from GLUT1‐IP lysate was decreased in Ishikawa cells with MGAT4A knockdown, and it was increased in HEC‐1B and SPEC‐2 cells with MGAT4A overexpression consistently. GLUT1 level in DSL‐IP lysate showed the similar results, suggesting a regulatory role for MGAT4A in GLUT1 glycosylation (Figure 4E). Furthermore, the interaction between GAL9 and GLUT1 glycoprotein was found to be dependent on MGAT4A by Co‐IP assay in Ishikawa cells (Figure 4F). Since GAL9 recognizes the galactose of LacNAc in carbohydrate chain and engagement galactose or lactose could impede its binding,^[^ ^40^ ^]^ we found that galactose, but not sucrose, substantially reduced the interaction between GLUT1 and GAL9 in Ishikawa cells (Figure 4F). In addition, GLUT1 N45D mutant abrogated GLUT1 and GAL9 interaction in 293T cells co‐transfected with MGAT4A and HA‐tagged GAL9 (Figure 4G). These results collectively highlighted MGAT4A's essential role in facilitating the glycosylation‐dependent interaction between GLUT1 and GAL9.

As GLUT1 undergoes continuous endocytosis and recycling, its membrane location is essential for efficient glucose uptake.^[^ ^41^ ^]^ We observed that GLUT1 N45D mutant decreased its presence on plasma membrane comparing to GLUT1‐WT in 293T cells (Figure 4H), indicating *N‐*glycan modification on this residue of GLUT1 protein for its intracellular distribution. Moreover, an augmented location of GLUT1 on cell membrane was noted when GAL9 was overexpressed in Ishikawa cells, and was diminished when MGAT4A was suppressed even in presence of GAL9 (Figure 4I). Subsequent pulse‐chase assay was conducted to measure the endocytosis of Flag‐tagged GLUT1 in the presence of GAL9 or empty vector (EV) in 293T cells (Figure 4J). The recycling of membrane‐bound Flag‐GLUT1 was hindered in GAL9 overexpressed cells (Figure 4K), supporting the hypothesis that GAL9 binding to GLUT1's *N‐*glycan impedes its endocytosis, thereby sustaining its availability on the plasma membrane.

Altogether, these data demonstrated a coordinated regulation between GAL9 and MGAT4A, which was indispensable for GLUT1 membrane localization in EC cells.

### 2.5 MGAT4A and GAL9 Co‐Promoted Cell Proliferation and Invasion Through GLUT1‐Mediated Glucose Metabolism

**Figure 5:**
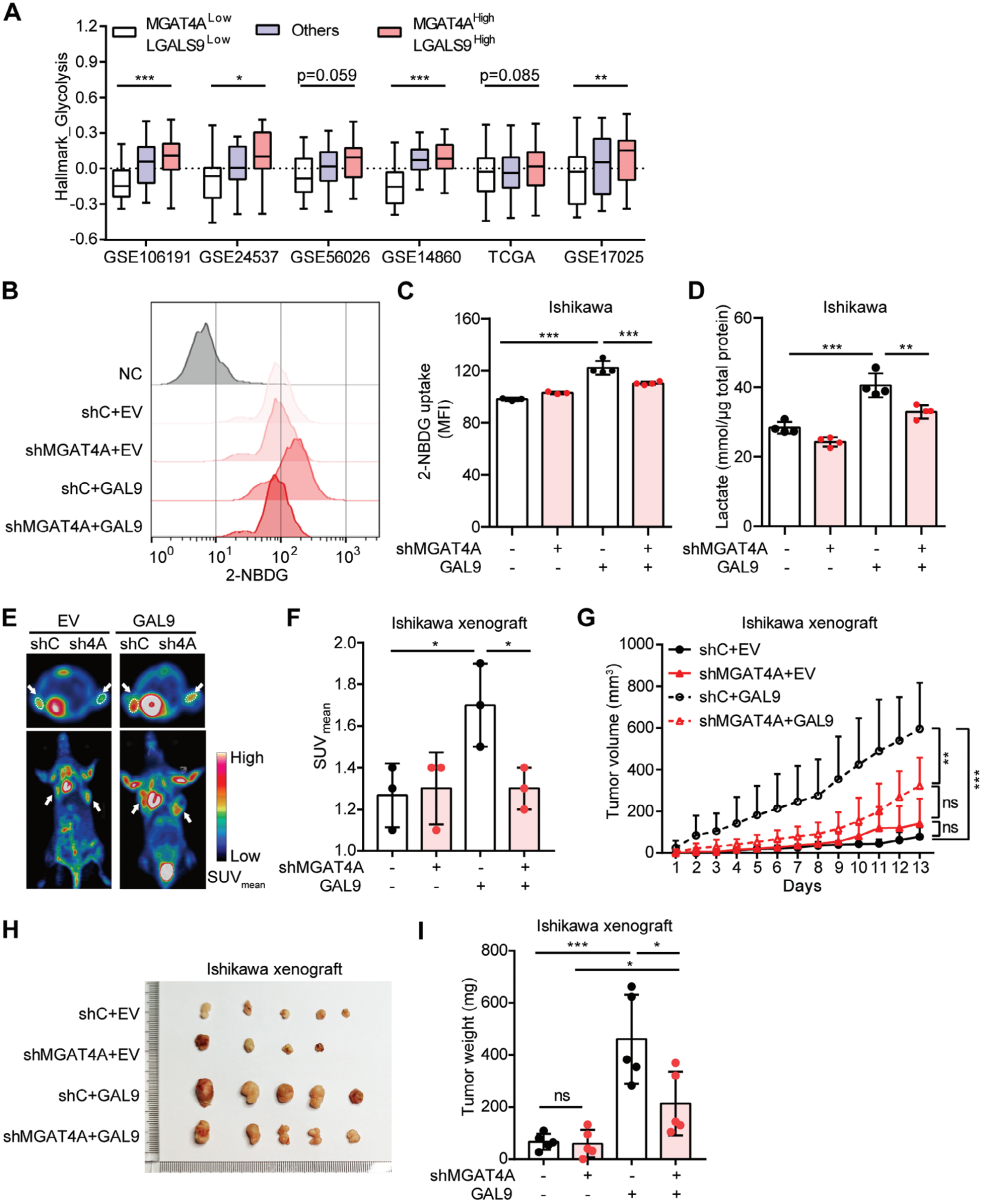
MGAT4A and GAL9 co‐promoted glucose metabolism in EC cells. A) The association between glycolysis signature score (Hallmark_Glycolysis) and MGAT4A/LGALS9 mRNA expression level in six public EC cohorts. B,C) 2‐NBDG uptake in Ishikawa cells with MGAT4A knockdown (shMGAT4A) or GAL9 overexpression (B), quantified by MFI of 2‐NBDG (C). D) Lactate production in Ishikawa cells with MGAT4A knockdown or GAL9 overexpression after 48 h culture. E) The representative axial and coronal ^18^F‐FDG PET images of Ishikawa xenografts‐bearing mice with MGAT4A knockdown or GAL9 overexpression. Xenografts were indicated by white arrows. F) SUVmean from different Ishikawa xenografts (E) were shown. G–I) Tumor growth curve (G), tumor photos (H), and tumor weight (I) of Ishikawa cells with MGAT4A knockdown or GAL9 overexpression. *p*‐values were calculated with one‐way ANOVA (A and G) and two‐way ANOVA (C, D, F, and I). ^*^ *p* < 0.05; ^**^ *p* < 0.01; ^***^ *p* < 0.001; ns, *p* ≥ 0.05.

To investigate whether MGAT4A and GAL9 were involved in glucose metabolism in EC patients, their associations with glycolysis signature were analyzed across six independent public EC datasets. Three different glycolysis signatures, including Hallmark_Glycolysis (GSEA hallmark gene sets), KEGG_Glycolysis_Gluconeogenesis, and ProteinAtlas glycolysis (The Metabolic Atlas Glycolysis pathway) were analyzed, respectively. EC patients with MGAT4A^High^LGALS9^High^ expression exhibited significantly higher signature scores for Hallmark_Glycolysis, compared to those with MGAT4A^Low^LGALS9^Low^ expression in four out of six datasets (**Figure** **5**A). The similar results were also observed for KEGG_Glycolysis_Gluconeogenesis signature and ProteinAtlas glycolysis signature (Figure S4A, Supporting Information). A synergistic regulation of MGAT4A‐GAL9 on glucose metabolism was suggested in EC patients.

In vitro glucose uptake and lactate production assays were therefore carried out. In Ishikawa cells with endogenous high MGAT4A, additional overexpression GAL9 significantly increased the uptake of 2‐NBDG (2‐(*N*‐(7‐nitrobenz‐2‐oxa‐1,3‐diazol‐4‐yl)amino)‐2‐deoxyglucose; an analog of glucose) (*p* < 0.001; Figure 5B,C), along with the lactate production (*p* < 0.01; Figure 5D); knockdown MGAT4A alone could diminish above effects (*p* < 0.01; Figure 5B–D). On the other hand, in SPEC‐2 cells with endogenous low MGAT4A, co‐overexpression of MGAT4A and GAL9 could remarkably increase both glucose uptake (*p* < 0.01; Figure S4B,C, Supporting Information) and lactate production (*p* < 0.01; Figure S4D, Supporting Information). In vivo glucose uptake was assessed by ^18^F‐FDG positron emission tomography (PET)/CT imaging. We observed a substantial increase in glucose uptake (*p* < 0.05; Figure 5E,F) and tumor growth (*p* < 0.001; Figure 5G–I) in Ishikawa xenografts with ectopic expression of GAL9; while MGAT4A inhibition reversed these effects (*p* < 0.05; Figure 5E–I). Besides, Western blot analyses showed no significant changes in component protein levels of mitochondrial complex I (NDUFB8), complex II (SDHA), complex III (UQCRC2), complex IV (COX4), and complex V (ATP5A), upon the overexpression or knockdown of MGAT4A/GAL9 in EC cells (Figure S4E, Supporting Information), indicating mitochondrial biogenesis and oxidative metabolism were not the key outcomes. Given that lactate production was significantly increased in MGAT4A and GAL9 high expressed cell (Figure 5D and Figure S4D, Supporting Information), suggesting that the increased glucose intake might be directed toward glycolysis pathway.

**Figure 6:**
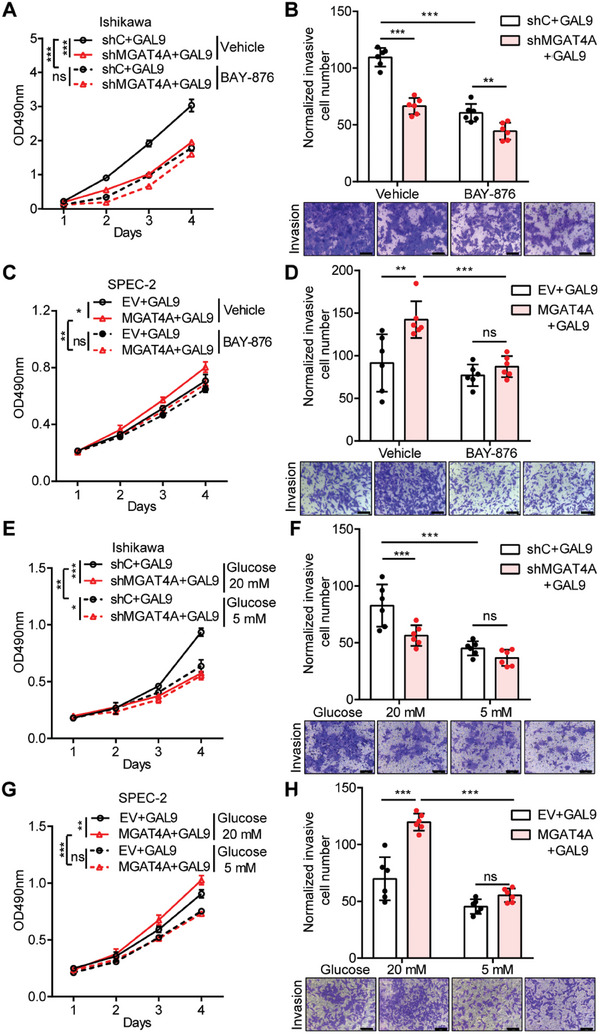
Cell proliferation and invasion co‐promoted by MGAT4A and GAL9 were dependent on GLUT1 activity and high level of glucose in EC cells. A,B) Cell proliferation by MTT assay (A) and cell invasion by Transwell assay (B) in GAL9 overexpressed Ishikawa cells, with MGAT4A knockdown (shMGAT4A and shC) or with GLUT1 inhibitor BAY‐876 treatment. C,D) Cell proliferation (C) and cell invasion (D) in MGAT4A and GAL9 co‐overexpressed SPEC‐2 cells with BAY‐876 treatment. E,F) Cell proliferation (E) and cell invasion (F) in GAL9 overexpressed Ishikawa cells with MGAT4A knockdown, under high‐glucose (20 mmol L^−1^ cell invasion (H) in MGAT4A and GAL9 co‐overexpressed SPEC‐2 cells, under high‐ and low‐glucose condition, respectively. Scale bar, 100 µm. *p*‐values were calculated with two‐way ANOVA. ^*^ *p* < 0.05; ^**^ *p* < 0.01; ^***^ *p* < 0.001; ns, *p* ≥ 0.05.

Next, we tested whether inhibition of glucose metabolism could suppress MGAT4A/GAL9‐driven cell proliferation and invasion. BAY‐876,^[^ ^42^, ^43^ ^]^ a GLUT1 specific inhibitor, was used in Ishikawa (2 µmol L^−1^) and SPEC‐2 cells (0.01 µmol L^−1^), respectively. Treatment with BAY‐876 significantly reduced GAL9‐mediated cell proliferation and invasion in Ishikawa cells (*p* < 0.001), and it had less effects on MGAT4A‐knockdown Ishikawa cells (**Figure** **6**A,B). Consistently, BAY‐876 treatment reduced the proliferation and invasion of SPEC‐2 cells co‐overexpressing MGAT4A and GAL9 (*p* < 0.01), and it had less effects on SPEC‐2 cells with GAL9 overexpression alone (Figure 6C,D). Considering that hyperglycemia is known to elevate the EC risk, potentially through augmenting GLUT1 activity and facilitating passive glucose transport,^[^ ^44^, ^45^, ^46^ ^]^ we hypothesized that normoglycemic condition (approximately 5 mmol L^−1^ glucose) might attenuate MGAT4A/GAL9‐driven cellular processes compared to hyperglycemic condition (approximately 20 mmol L^−1^ glucose). Decreased glucose level had the similar effects as BAY‐876 treatment only on proliferation and invasion of Ishikawa with GAL9 overexpression (*p* < 0.01; Figure 6E‐F) and SPEC‐2 cells with MGAT4A and GAL9 co‐overexpression (*p* < 0.001; Figure 6G,H).

These findings suggested that the pro‐proliferative and invasive effects by MGAT4A/GAL9 were partially relying on GLUT1‐mediated glucose uptake.

### 2.6 MGAT4A and GAL9 Co‐Promoted Tumor Aggressiveness Through Mediating GLUT1 Membrane Location in EC Patients

**Figure 7:**
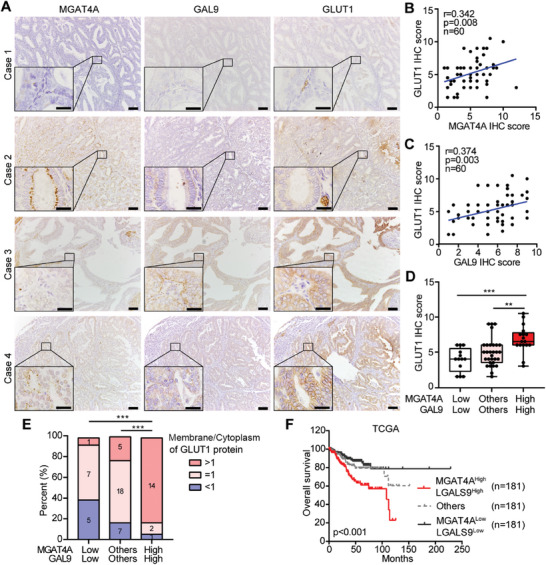
MGAT4A and GAL9 co‐promoted tumor aggressiveness through mediating GLUT1 membrane location in EC patients. A) The representative IHC images for MGAT4A, GAL9 and GLUT1 in human EC patients from our cohort (*n* = 60). Case 1, MGAT4A^Low^GAL9^Low^; Case 2, MGAT4A^High^GAL9^Low^; Case 3, MGAT4A^Low^GAL9^High^; Case 4, MGAT4A^High^GAL9^High^. Scale bar, 200 µm; inset, 20 µm. B, C) The associations of MGAT4A protein (B) and GAL9 protein (C) with GLUT1 protein in human EC patients from our cohort, respectively. D,E) The associations between MGAT4A‐GAL9 and total GLUT1 protein (D) or membrane‐bound GLUT1 protein (E) in human EC patients from our cohort, respectively. F) Kaplan–Meier plot based on overall survival of EC patients from TCGA‐UCEC cohort, stratified by MGAT4A‐LGALS9 mRNA level. *p*‐values were calculated with one‐way ANOVA (D) and χ^2^ test (E). ^**^ *p* < 0.01; ^***^ *p* < 0.001.

The clinical correlations among MGAT4A, GAL9 and GLUT1 in human EC specimens were analyzed using their IHC scores (**Figure** **7**A). Both MGAT4A and GAL9 showed the positive associations with GLUT1 protein expression (*r *> 0.3, *p* < 0.01; Figure 7B,C). For EC patients with MGAT4A^High^GAL9^High^ IHC score, their GLUT1 protein expression was higher than other patients (*p* < 0.01; Figure 7D); particularly for the membrane‐bound GLUT1 protein (*p* < 0.001; Figure 7E). We also analyzed the EC patients in TCGA‐UCEC dataset, overall survival of MGAT4A^High^LGALS9^High^ group was lower than other groups (*p* < 0.001; Figure 7F). A synergistic regulation of MGAT4A‐GAL9 on GLUT1 membrane location promoting tumor aggressiveness was suggested in EC patients, aligning with their effects on glucose uptake for glucose metabolism in vitro and in vivo.

### 2.7 p53 as a Negative Regulator of MGAT4A via miR‐34a and miR‐449a/b

**Figure 8:**
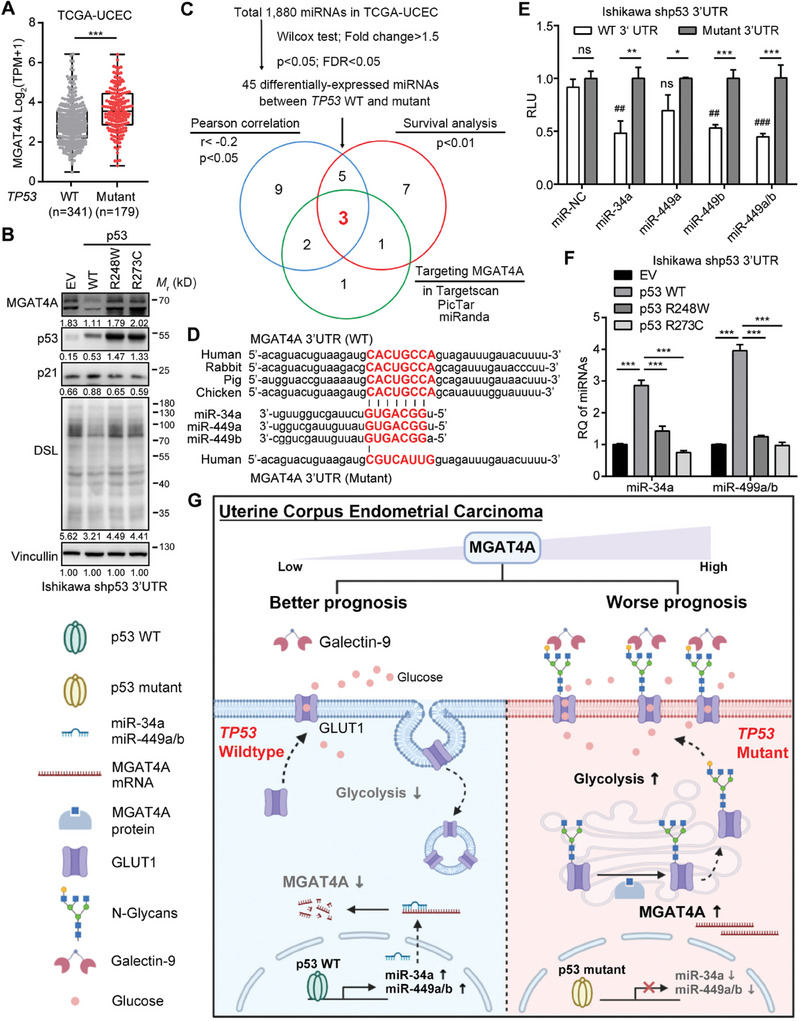
A regulatory axis involving p53/miRNAs/MGAT4A in EC. A) Association between *TP53* genetic alteration and MGAT4A mRNA level in EC patients from TCGA‐UCEC cohort. B) MGAT4A protein and the levels of β‐1,4 linked *N*‐acetylglucosamine oligomers mediated by WT and mutated p53 (R248W and R273C) in Ishikawa cells using Western blot analyses. C) Screening process for the p53‐regulated miRNAs targeting MGAT4A in EC patients from TCGA‐UCEC cohort. D) The predicted targeting sequences of miR‐34a, 449a, and 449b on MGAT4A 3’UTR across different species. E) The relative luciferase activity of MGAT4A 3’UTR reporters in Ishikawa cells transfected with miR‐34a, 449a, 449b, 449a/b, or miR‐NC control. ^##^ *p* < 0.01; ^###^ *p* < 0.001; comparison between miRNA and miR‐NC. F) Expression levels of miR‐34a and miR‐449a/b upon WT or mutated p53 overexpression in Ishikawa cells. G) The simplified working model for a novel regulatory axis involving p53/miRNAs/MGAT4A‐GAL9 on GLUT1‐dependent glucose metabolism in EC patients with *TP53* mutations, which have worse prognosis. *p*‐values were calculated with unpaired two‐tail Student's *t*‐test (A and E) and one‐way ANOVA (F). ^*^ *p* < 0.05; ^**^ *p* < 0.01; ^***^ *p* < 0.001; ns, *p* ≥ 0.05.

p53 is a critical tumor suppressor known for its regulation of cancer cell metabolism, and it has been reported to influence protein *N*‐glycosylation.^[^ ^47^, ^48^, ^49^ ^]^ Our observations corroborated this finding, showing a significant upregulation of MGAT4A in EC patients harboring *TP53* mutations in the TCGA‐UCEC dataset (*p* < 0.001; **Figure** **8**A). To detect the effects of p53 mutant variants on EC cells, endogenous p53 expression in Ishikawa cells was stably knocked down using shRNA targeting a 3’UTR region of p53 mRNA (Ishikawa shp53 3’UTR; Figure S5A, Supporting Information). Then, two hotspot mutations (R248W or R273C) and WT p53 were stably transduced into Ishikawa shp53 3’UTR cells, respectively (Figure 8B). Both R248W and R273C mutants could increase MGAT4A protein levels and DSL binding (Figure 8B), as well as GLUT1 protein levels, membrane localization, and 2‐NBDG uptake in the CM from GAL9‐overexpressing Ishikawa cells (GAL9‐CM) (Figure S5B–D, Supporting Information), comparing to WT p53.

Moreover, to investigate whether tumor aggressiveness and abnormal glucose metabolism induced by p53 mutants were MGAT4A‐dependent in EC cells, siRNA targeting MGAT4A was used to inhibit MGAT4A expression in Ishikawa shp53 3’UTR cells (Figure S5E, Supporting Information). In GAL9‐CM, R273 mutant could enhance 2‐NBDG uptake (*p* < 0.01), cell viability (*p* < 0.01), and invasiveness (*p* < 0.001), compared to WT p53; and siMGAT4A treatment significantly suppressed 2‐NBDG uptake (*p* < 0.05), cell viability (*p* < 0.001), and invasiveness (*p* < 0.001) in cells with R273 mutant cells, but not in cells with WT p53 (Figure S5F–H, Supporting Information). These findings supported the notion that mutant p53 enhanced GLUT1 function through MGAT4A/GAL9‐mediated aberrant *N*‐glycosylation, contributing to altered glucose metabolism in cancer cells.

Thus, we explored whether p53‐regulated miRNAs could target MGAT4A mRNA, as no consensus p53 binding site was identified in the MGAT4A gene promoter using bioinformatics tools and public ChIP‐seq data. In TCGA‐UCEC dataset, 45 miRNAs were found differentially expressed in *TP53* mutation from those in *TP53* WT patients (Fold change>1.5, *p* < 0.05; FDR<0.05). Further analyses using three criteria were performed, 1) miRNA should contain the predicted binding motif to MGAT4A mRNA; 2) miRNA level should negatively correlate to MGAT4A mRNA level (r < ‐0.2, *p* < 0.05); and 3) miRNA level should correlate to favorable prognosis (*p* < 0.01) (Figure 8C). Three miRNAs (miR‐34a, miR‐499a, and miR‐499b) were identified with a common seed sequence targeting MGAT4A (Figure 8D). 3‐miRNA expression signature score showed a significant inverse correlation with MGAT4A expression (*r* = −0.3354, *p* < 0.001; Figure S6A, Supporting Information), advanced tumor stage (*p* < 0.05; Figure S6B, Supporting Information) and good clinical outcome (*p* = 0.0004; Figure S6C, Supporting Information) in TCGA‐UCEC cohort. Transfection of miR‐34a and miR‐499a/b mimics into Ishikawa cells reduced MGAT4A protein expression (Figure S6D, Supporting Information), β1,4‐GlcNAc modification (Figure S6D, Supporting Information), and the reporter activity of MGAT4A 3’UTR (Figure 8E). The expression levels of these three miRNAs were elevated by WT p53 but not by mutant p53 (Figure 8F); meanwhile, miR‐34a and miR‐499a/b inhibited cell invasion (Figure S6E, Supporting Information), cell proliferation (Figure S6F, Supporting Information), and lactate production (Figure S6G, Supporting Information) in Ishikawa cells with GAL9 overexpression, highlighting the negative regulation of p53/three‐miRNAs on MGAT4A expression for glucose metabolism and tumor aggressiveness.

In summary, our results revealed a novel regulatory axis involving p53/miRNAs/MGAT4A‐GAL9 on GLUT1‐dependent glucose metabolism in EC patients with *TP53* mutations, which have worse prognosis. In these patients, p53 mutants could not activate the transcription of miR‐34a and miR‐499a/b, resulted in the repression loss of MGAT4A post‐transcription. The overexpressed MGAT4A with GAL9 collaboration further increased the *N‐*glycan modification and membrane localization of GLUT1 to promote glycolysis for tumor proliferation and invasion (Figure 8G). How the genetic alteration of *TP53* promoted tumor aggressiveness via multiple regulatory means, including miRNA, glycosylation, subcellular localization, and metabolism, on the MGAT4A‐related signaling axis, has been demonstrated in EC.

## 3 Discussion

Aberrant glycosylations have been implicated in the aggressiveness of many cancer types.^[^ ^50^ ^]^ Though some tumor biomarkers derived from abnormal glycoforms, such as CA153, have been used to distinguish normal and EC samples,^[^ ^51^ ^]^ the roles of glycogenes in EC progression are not extensively or systematically investigated. In this study, we successfully identified that glycosyltransferase MGAT4A was a potential biomarker for distinguishing poor prognostic EC subtype and characterized its tumor promoting role in EC development via reprogramming glucose metabolism, as evidenced below: 1) An unsupervised clustering analysis identified an 11‐glycogene cluster, which was characterized with poor prognosis and enriched with *TP53* mutation, using the public EC dataset TCGA‐UCEC (*n* = 543). Among this novel cluster, MGAT4A was the most representative glycogene, whose overexpression was significantly associated with poorly differentiated histological subtype and adverse prognosis in EC patients, which was further validated in EC samples from our own cohort (*n* = 60). 2) MGAT4A upregulation promoted EC aggressiveness in vitro and in vivo, which required the presence of Galβ1‐4GlcNAcβ reader GAL9. 3) *N‐*glycoproteomics revealed GLUT1 protein was one of the major glycoproteins modified by MGAT4A. The binding of GAL9 to the MGAT4A‐branched *N‐*glycan on GLUT1 protein prolonged GLUT1 on cell membrane, leading to glucose uptake increase. 4) We revealed the indirect downregulation of MGAT4A by WT p53 via three tumor suppressive miRNAs, miR‐34a, miR‐449a, and miR‐449b. In the EC with *TP53* mutations, p53 mutants lost such tumor suppressive function on MGAT4A expression and resulted in poor prognosis of patients. These findings underpinned the notion that centered on MGAT4A, p53‐mutants promote endometrial carcinoma (EC) aggressiveness through miRNAs, *N‐*glycosylation, and glucose metabolism.

MGAT4A belongs to the branching *N*‐acetylglucosaminyltransferase family, responsible for the trimming and rebuilding *N‐*glycans in Golgi apparatus to produce tissue‐ and disease‐specific *N‐*glycans. Among the MGAT family proteins, namely MGAT1, 2, 3, 4A/B/C and 5, MGAT4A, and MGAT4B are isozymes to catalyze the transfer of GlcNAc to α1‐3‐linked mannose of the core structure of *N‐*glycan via β1‐4 linkage, while such activity of MGAT4C awaits validation. Due to its high affinity for donor and acceptor substrates, MGAT4A, but not MGAT4B, is regarded as the primary enzyme for complex‐type *N‐*glycans formation.^[^ ^52^ ^]^ In addition, the expression patterns of MGAT4A and MGAT4B are not the same in mammalian tissues. For example, MGAT4A expression was high in gastrointestinal tissues, while MGAT4B was ubiquitously expressed.^[^ ^53^ ^]^ In pancreatic cancer, MGAT4A was downregulated due to the promoter methylation, while MGAT4B was overexpressed.^[^ ^54^ ^]^ Based on the IHC data from Human Protein Atlas endometrial cancer database, MGAT4A was overexpressed mainly in EC cells and MGAT4B was expressed in tumor stroma (Figure S2I, Supporting Information). Taken together, MGAT4A does not function redundantly as MGAT4B under physiological and pathological conditions.

Notably, we observed some discrepancies in the growth curves between Ishikawa cells (Figure 3C) and Ishikawa xenografts (Figure 5G). MGAT4A knockdown in Ishikawa cells (with low GAL9) suppressed cell growth in vitro (*p* < 0.001); however, xenografts from Ishikawa cells, with or without MGAT4A knockdown, grew very slow (*p* ≥ 0.05), indicating that MGAT4A could not significantly drive xenograft growth alone without GAL9. It was also shown the minor difference between shMGAT4A+GAL9 and shMGAT4A+EV groups in vitro, despite *p* < 0.01; and there was no significant difference between these two groups in vivo, indicating that without MGAT4A, GAL9 could not significantly modulate tumor cell growth alone. These discrepancies between in vitro and in vivo may attribute to the interaction between tumor cells and tumor microenvironment, along with other dysregulated glycogenes. As a versatile secretory protein, GAL9 might affect not only tumor cells but also various components of the tumor stroma, including TIM‐3‐mediated T cell death, M2 polarization of macrophage, and expansion of myeloid‐derived suppressor cells.^[^ ^55^, ^56^, ^57^ ^]^ On the other hand, dysregulated glycotransferases in tumor cells may also regulate *N*‐glycosylation of the membrane proteins to reduce tumor surveillance. For example, B3GNT3 catalyzed the glycosylation on PD‐L1 in triple‐negative breast cancer cells, while MYC‐induced ST6GALNAC4 overexpression led to Siglec‐directed immune suppression.^[^ ^58^, ^59^ ^]^ Recent paper also reported that the loss of MGAT3, another MGAT family member, promoted tumor‐associated changes in *N*‐glycosylation, destabilize IFNγRα, causing IFN‐γ resistance in colorectal cancer cells.^[^ ^60^ ^]^ Furthermore, given that GAL9 preferentially recognizes LacNAc residue, which is not the direct product of MGAT4A, indicating that other glycogenes related to the production of this residue might be also dysregulated in Ishikawa xenografts. Altogether, it was implied that GAL9 overexpression‐induced growth increase of Ishikawa cell/xenograft was partially dependent on the presence of MGAT4A.

The dysregulation of MGAT4A in various cancer types suggests that identification of MGAT4A's substrates may account for its tumor‐promoting role. In choriocarcinoma, integrin β1 was modified by MGAT4A to promote tumorigenicity.^[^ ^61^ ^]^ Herein, we exploited the *N*‐glycoproteomics approach and demonstrated GLUT1 as a substrate of MGAT4A. The *N*‐glycans on GLUT1 catalyzed by MGAT4A provided the platform for further interaction between GLUT1 and GAL9 to reduce the turnover of GLUT1 protein on plasma membrane. Our data indicated one paradigm for the *N*‐glycan/galectin lattice model, i.e. the branched *N*‐glycans could enhance the interaction of membrane proteins, such as receptors, integrins, and solute carrier transporters, with different galectins, thereby prolonging the duration of glycoprotein on cell membrane and remaining their functions and/or activation of downstream signaling pathways.^[^ ^61^, ^62^ ^]^ Particularly, such *N*‐glycan/galectin lattice could also re‐distribute the membrane glycoprotein into or out of lipid‐raft microdomain.^[^ ^63^ ^]^

Glucose metabolism reprogramming has been implicated in malignant transformation of normal endometrial cells and EC development.^[^ ^28^ ^]^ As a key glucose transporter in aerobic glycolysis, GLUT1 plays a critical role in EC aggressiveness.^[^ ^64^, ^65^, ^66^ ^]^ During carcinogenesis, GLUT1 is delicately regulated by a plethora of factors, including transcription factors (p53 and HIF1α) and transcription cofactor (SRC‐3) at transcriptional level,^[^ ^67^, ^68^, ^69^ ^]^ regulators for mRNA stability (miR‐378a and Mettl3),^[^ ^70^, ^71^ ^]^ as well as regulators for protein stability (GAL, a GLUT1‐associated lncRNA).^[^ ^72^ ^]^ Previous work also reported that increased *N‐*glycan modification promoted GLUT1 stability and enhanced GLUT1 function and impairment of *N‐*glycosylation of GLUT1 resulted in disruption of GLUT1 clustering and glucose uptake.^[^ ^73^, ^74^ ^]^ Herein our study provided another perspective that MGAT4A/GAL9 cooperatively promoted GLUT1 localization on cell surface through *N‐*linked glycosylation.

Frequent *TP53* mutation is found in aggressive EC subtype with poor prognosis; however, few mechanistic insights have been reported. Our data indicated that p53 mutants (R248W and R273C) promoted glucose metabolism through GLUT1 *N‐*glycosylation mediated by upregulating MGAT4A mRNA level, through the transcription regulation of miR‐34a and miR‐449a/b. Among them, miR‐34a is a p53 target miRNA,^[^ ^75^ ^]^ and miR‐449a and ‐449b are responsive to WT p53, but not its mutated forms. In addition to miRNAs, which usually bind to 3’UTR of the target mRNAs to suppress their expression, there may exist other non‐coding RNAs‐associated regulatory mechanisms. For certain miRNAs, such as miR‐146a‐5p, can activate gene expression through binding to the gene enhancer.^[^ ^76^ ^]^ tRNA‐derived fragments (tRFs), long non‐coding RNAs and circRNAs have also been shown to play significant roles in gene expression.^[^ ^77^, ^78^ ^]^ tRFs can influence gene translation and RNA stability, whereas lncRNAs or circRNA can act as molecular sponges for miRNAs, scaffolds for protein complexes, or direct transcriptional regulators. Further studies are needed to explore more regulations of non‐coding RNAs on MGAT4A gene expression and their interactions with p53 mutations, which might provide the deeper insights into mechanisms underlying the malignant progression of EC with *TP53* mutation. Moreover, the potential mutual exclusion between *TP53* and *PTEN* mutation was observed in EC patients from TCGA‐UCEC dataset (Figure 1A), how PTEN gene alterations promote EC should be explored in subsequent study.

Overall, this study identified a *TP53* mutation‐EC subtype with increased glucose metabolism for tumor progression. This molecular EC subtype relied on MGAT4A/GAL9‐mediated GLUT1 protein modification, promoting glucose metabolism for tumor proliferation and invasion. Since we investigated mRNA expression levels of glycogenes from public EC transcriptome data for the key regulators of *N‐*glycosylation, we could only identify the ones with significant transcriptional changes in EC patients using this indirect searching strategy. Proteomic and *N‐*glycoproteomic analyses on the EC samples will be performed to directly identify the regulators and the substrates of abnormal *N‐*glycosylation. Moreover, direct blocking MGAT4A/GLUT1 activity or indirect interference in GLUT1 *N‐*glycosylation are suggested as potential therapeutic strategies for EC patients.

## 4 Experimental Section

### Data Retrieval from the Public Databases

Data of mRNA expression, protein expression, gene mutation, and clinical outcome from EC patients were sourced from The Cancer Genome Atlas (TCGA) via the Genomic Data Commons Data Portal (https://portal.gdc.cancer.gov/). The FPKM (fragments per kilobase of transcript per million mapped reads) data were normalized to TPM (transcripts per million) to facilitate inter‐sample comparisons. The normalization was achieved using the formula:

(1) $$\mathrm{TPM}=\frac{\mathrm{FPKM}}{\sum \left(FPKM\right)/1,000,000}$$

Other public EC databases were obtained from the Gene Expression Omnibus (GEO) (http://www.ncbi.nlm.nih.gov/geo). For multiple probes, the mean expression level was computed and utilized for further analysis. Corresponding clinical information was also retrieved.

Mature strand miRNA expression data were downloaded from the UCSC Xena platform (http://xena.ucsc.edu), where expression values were already transformed and represented as Log_2_(1+RPM).

The IHC results of MGAT4A&B were retrieved from the Human Protein Atlas database (https://www.proteinatlas.org/).

### Hierarchical Clustering and Gene Expression Analysis

A comprehensive set of 210 glycogenes was collated from the GlycoGene DataBase (GGDB, https://acgg.asia/ggdb2/) and supplemented by data from a prior study.^[^ ^47^ ^]^ These glycogenes underwent annotation using the DAVID 6.8 bioinformatics tool (https://david.ncifcrf.gov/tools.jsp), resulting in a refined list of 195 unique glycogenes. From this list, 145 glycogenes, showing expression in more than 20% tissue samples with TPM > 1 and an average TPM > 1, were selected for further analysis. Unsupervised hierarchical clustering of TCGA‐UCEC cohort was executed using the Morpheus tool (https://software.broadinstitute.org/morpheus) and the Average Linkage method with One minus Pearson correlation. Differential gene expression across the identified clusters was analyzed using the Limma R‐package, specifically focusing on genes that exhibited significant changes (Log_2_Fold Change>1, *p*‐value < 0.05, and FDR (*q*‐value) < 0.05).

### Patient Sample Collection and Study Approval

The Institutional Review Board committee of Shanghai General Hospital approved collection of the EC samples and corresponding clinicopathological data, such as age, tumor stage, tumor grade, tumor histology, ER/PR expression, and recurrence status. All relevant ethical regulations for work with human participants had been compiled and written informed consents were obtained. The project number was 2019SQ199.

### IHC Staining

IHC was performed as previously described.^[^ ^24^ ^]^ The antibodies for IHC were listed in Table S4 (Supporting Information). Semiquantitative optical analysis was utilized to calculate staining scores. The score combined the percentage of positive cells (graded as 1 (0–25%), 2 (25–50%), 3 (50–75%), and 4 (75–100%)) with staining intensity (graded as 1 for weak, 2 for moderate, and 3 for strong). The final score, ranging from 1 to 12, was determined by multiplying the percentage staining score with the intensity score. To assess the membrane‐bound GLUT1 protein, the ratio of mean membrane signal intensity to mean cytoplasm signal intensity was used.

### Cell Culture and Reagents

Human Ishikawa, HEC‐1B and AN3CA cell lines were obtained from Cell Bank of Type Culture Collection, Chinese Academy of Sciences (Shanghai, China). HEC‐1A cell line was purchased from ATCC (American Type Culture Collection, Manassas, VA). SPEC‐2 cell line was purchased from Ranqi Biological Technology (Shanghai, China). Ishikawa, AN3CA, HEC‐1A, and HEC‐1B cells were maintained in DMEM, SPEC‐2 cells were cultured in RPMI 1640 medium, supplemented with 10% fetal bovine serum (10099, Thermo Fisher Scientific, Australia) plus 100× penicillin and streptomycin (30‐002‐CI, Corning, NY). For experiments involving glucose uptake and varying glucose conditions culture, cells were cultured in a glucose‐free medium (11966, Thermo Fisher Scientific). Cells were incubated at 37 °C in an atmosphere of 5% CO_2_ and 95% air. Lentiviral particles for MGAT4A knockdown (shMGAT4A), MGAT4A overexpression, and galectins overexpression were generated from 293T cell, followed by the transduction to Ishikawa, HEC‐1B and SPEC‐2 cells, respectively. Cells were selected in 1 µg mL^−1^ puromycin (P8833, Sigma‐Aldrich, St. Louis, MO) or blasticidin S (SBR00022, Sigma‐Aldrich) for 5 days. Chemicals and kits were listed in Table S4 (Supporting Information). The subcloning sequences for each plasmid were listed in Tables S5 and S6 (Supporting Information).

### Western Blotting Analysis

Total proteins were extracted from frozen tissues or cultured cells using RIPA buffer supplemented with PhosSTOP (4906845001, Roche, Indianapolis, IN), cOmplete (11836170001, Roche), and 1% (v/v) Triton X‐100. For GLUT1 blotting, samples were denatured at 37 °C for 30 min in 1 × Laemmli sample buffer containing 50 mmol L^−1^ dithiothreitol. For lectin blotting, 1 × Carbo‐Free blocking solution (SP‐5040, Vector Laboratories, Burlingame, CA) was used to block the nonspecific binding and subsequently incubated with DSL at 4 °C overnight. After TBST washing, the membranes were incubated with Streptavidin‐HRP for 1 h and detected with the enhanced chemiluminescence reagents. The antibodies were listed in Table S4 (Supporting Information).

### IF Staining

Cells were fixed with 4% paraformaldehyde and permeabilized with 0.5% Triton X‐100 for 20 min. For DSL staining, cells were blocked with Carbo‐Free blocking solution. For GLUT1 or HA‐tag staining, cells were blocked with 5% non‐fat milk for 1 h. Then, cells were subsequently incubated with DSL or primary antibodies at 4 °C overnight. After washing, cells were incubated with fluorescent secondary antibody for 1 h. DAPI counterstained the nuclei for 5 min. Images were acquired using a LSM880 Laser confocal microscopy (ZEISS, Germany). Antibodies were listed in Table S4 (Supporting Information).

### Cell Proliferation and Invasion Assays

For cell proliferation assay, 2 × 10^3^ cells per well were seeded into 96‐well plates. Cell viability was determined everyday by MTT (3‐(4,5‐dimethylthiazol‐2‐yl)‐2,5‐diphenyltetrazolium bromide) over 4 days. For cell invasion assay, the Transwell inserts (8 µm) were precoated with Matrigel (354165, Corning) at 37 °C for 1 h. A total of 1×10^5^ serum‐starved cells were seeded on top of Transwell inserts. After 36 h incubation, cells were fixed with 4% paraformaldehyde and stained with 0.2% crystal violet. Non‐invading cells were removed with cotton swab, and the invaded cells were counted. Considering the influence of cell proliferation on the invaded cell number, it was normalized by proliferation rate of the corresponding cells using MTT assay. All experiments were repeated at least 3 times.

### DSL IP and Cross‐Link co‐IP

Ishikawa, HEC‐1B, and SPEC‐2 cells expressing the indicated constructs were lysed with Buffer A (40 mmol L^−1^ HEPES [pH 7.5], 120 mmol L^−1^ NaCl, 1 mmol L^−1^ EDTA, 10 mmol L^−1^ pyrophosphate, 10 mmol L^−1^ glycerophosphate, 50 mmol L^−1^ NaF, 1.5 mmol L^−1^ Na_3_VO_4_, supplied with 1% Triton X‐100 and cOmplete). Supernatants of cell lysis were collected by centrifugation at 16 000 g for 30 min. An amount of total of 1 mg of each lysate was precleaned with 30 µL avidin agarose beads (20219, Thermo Fisher Scientific) and incubated with 10 µmol L^−1^ DSL lectin or 10 µmol L^−1^ biotin at 4 °C overnight. The next day, samples were incubated with 30 µL avidin agarose beads at 4 °C for 4 h prior to centrifugation at 5000 g and washed by Buffer A three times before Western blotting.

For GLUT1 and galectins interaction analysis, 1 × 10^7^ cells with Flag‐GLUT1 and HA‐galectins expression were cross‐linked with 50 mmol L^−1^ DSP (dithiobis(succinimidyl propionate); C110213, Sangon Biotech, Shanghai, China) for 30 min, and stopped with quench buffer (50 mmol L^−1^ Tris, 10 mmol L^−1^ EDTA, 150 mmol L^−1^ NaCl, pH 7.4) for 15 min at room temperature. Cross‐linked cells were lysed with Buffer A and scratched to harvest the cell lysate. Supernatants of cell lysates were collected by centrifugation at 16 000 g for 30 min. An amount of 1 mg of lysates were precleaned with 30 µL protein A/G beads (sc‐2003, Santa Cruz, Dallas, TX) and incubated with the anti‐GLUT1 or anti‐HA tag antibody at 4 °C overnight. The next day, samples were incubated with 30 µL of protein A/G beads at 4 °C for 4 h. The beads were washed three times with quench buffer containing 1% Triton X‐100. Elution was carried out in 30 µL of Laemmli sample buffer with 50 mmol L^−1^ dithiothreitol for 1 h at 37 °C to reduce the DSP cross‐linking prior to Western blotting. Antibodies were listed in Table S4 (Supporting Information).

### *N*‐glycoproteomic Analysis

Ishikawa cells with MGAT4A overexpression or knockdown were lysed with 600 µL RIPA buffer for 30 min on ice followed by sonication. After centrifugation, protein concentration was determined by BCA assay. Next, 500 µg protein lysates were precipitated with 10% TCA and washed with pre‐cold acetone. The pellet was then mixed thoroughly in 50 mmol L^−1^ NH_4_HCO_3_ buffer [pH 7.5]. Then, the samples were digested with sequencing‐grade modified trypsin (enzyme to protein ratio 1:50, w/w) at 37 °C overnight. The mixed samples were reduced with 5 mmol L^−1^ DTT at 56 °C for 30 min, alkylated with 11 mmol L^−1^ iodoacetamide at room temperature for 30 min in darkness. Samples were then acidified with formic acid (FA) and desalted. An amount of 200 µg of the desalted peptides were enriched using a HILIC‐based enrichment procedure.

Glycosylated peptides were dissolved in 0.1% FA and separated by an EASY‐nLC 1200 UHPLC system (Thermo Fisher Scientific) coupled to a Q Exactive HF‐X mass spectrometer (Thermo Fisher Scientific), then were injected into a reversed‐phase C18 column (20 cm length × 75 µm ID, 1.9 µm particle size, Dr. Maisch GmbH, Germany) in a 120 min gradient from 2% to 80% solvent B (80% acetonitrile in 0.1% FA). Full scan MS spectra (mass range from m/z 700 to 1800) were acquired in the Orbitrap with resolution of 120 000. The automatic gain control (AGC) was set as 3e6. The top 15 precursor ions were selected from each MS full scan. Subsequently, MS/MS spectra were acquired in the Orbitrap with resolution of 15 000. The AGC was 2e5 and the maximum injection time was 200 ms. Dynamic exclusion was set to 15.0 s.

DIA‐NN software (version 1.8) and PGlyco (version 3.0) were used to analyze the proteome and glycosylation raw data. Spectrum was extracted using Glyco‐Decipher (version 1.0.3). The MS/MS spectra were searched against UniProt human database (released in Jan 2020). Cysteine carbamidomethylation was established as a fixed modification, *N‐*terminal acetylation and methionine oxidation were established as variable modifications. FDR of 0.01 was required for proteins and peptides. Enzyme specificity was set to trypsin and two missed cleavage sites were allowed.

### Mapping Analysis for GLUT1 Protein *N*‐glycosylation

293T cells were co‐transfected with Flag‐GLUT1, MGAT4A, and HA‐GAL9 for 48 h. Cells were lysed and GLUT1 protein was immunoprecipitated by Flag‐beads (A2220, Sigma‐Aldrich). The protein was digested with sequencing‐grade modified trypsin (enzyme to protein ratio 1:50, w/w) at 37 °C overnight. Then, the sample was then acidified with FA and desalted.

An EASY‐nLC 1200 UHPLC system coupled to a Q Exactive HF‐X mass spectrometer was used for sample analysis. Peptides were separated with a gradient from 2% to 80% solvent B over 30 min. A total scanning was acquired from 700 to 1800 m/z, using a 120 000‐resolution precursor ion scan followed by MS/MS of the top 15 most intensive ions at 15 000 resolution. For the MS/MS analyses, the AGC number was 2e5 with a maximum ion injection time of 200 ms. Glycosylation raw data were analyzed by PGlyco (version 3.0) and MS/MS spectra were searched against UniProt human database.

### Flow Cytometry for Membrane‐Bound GLUT1

A total of 5 × 10^6^ 293T cells were transiently transfected with Flag‐GLUT1‐GFP and MGAT4A for 48 h. After trypsinization, cells were treated by CM from cells with GAL9 overexpression for 30 min. Subsequently, they were incubated with anti‐Flag M2 antibody at 37 °C for 30 min. Following the washing step, cells were returned to the regular culture medium for various durations (0, 30, 60, and 90 min) to facilitate GLUT1 internalization. The cells were then treated with Cy5 AffiniPure Donkey Anti‐Mouse IgG antibody recognizing Flag‐tag. Flow cytometry was performed using a BD LSRFortessa (BD Biosciences, San Jose, CA). The MFI (Mean Fluorescence Index) of Cy5 in GFP‐positive cells was analyzed at each time point. Relative membrane GLUT1 at each time point was normalized using the MFI at 0 min for each sample.

### Measurement of Glucose Uptake In Vitro

A total of 5 × 10^4^ cells were seeded in each well of 96‐well plates on the first day. Cells were treated with glucose‐free culture medium for 1 h, followed by the treatment of 10 µmol L^−1^ 2‐NBDG (N13195, Invitrogen, Waltham, MA) for 4 h. Cell fluorescence was measured using a BD FACSCalibur (BD Biosciences). MFI of 2‐NBDG was used for the glucose uptake activity.

### Lactate Production Measurement

A total of 5 × 10^6^ Ishikawa and SPEC‐2 cells were cultured in complete media. After 48 h, cells were scratched and homogenized with PBS and centrifuged with 13 000 g at 4 °C for 15 min. The total protein was quantified by BCA kit. l‐lactate was quantified using Lactic acid assay kit (A019‐2‐1, Nanjing Jiancheng Bioengineering Institute, China) using the manufacturer's protocol. The concentration of lactate in the samples was calculated based on the ratio of l‐lactate (mmol) to the total protein content (µg) measured in the samples.

### Measurement of Glucose Uptake In Vivo

All animal experiments were approved by the Institutional Animal Care & Use Committee of Shanghai Institute of Materia Medica, Chinese Academy of Sciences and adhered to the NIH Guide for the Care and Use of Laboratory Animals. The IACUC number is 2021‐08‐HRM‐47.

^18^F‐FDG uptake in vivo was evaluated by PET/CT imaging. A total of 4‐ to 6‐week‐old female athymic nude mice were purchased from HFK BIOSCIENCE (Beijing, China). Approximately 5 × 10^6^ Ishikawa cells, stably infected with shMGAT4A or shC, along with GAL9 or EV expressing lentiviral particles, were subcutaneously injected into both flanks of the mice with 50% matrigel (354234, Corning), grouped as shMGAT4A/EV and shC/EV, or shMGAT4A/GAL9 and shC/GAL9 in one mouse. Four weeks later, the tumor‐bearing mice were fasted for 8 h before injection of ^18^F‐FDG (100 to 200 µCi) via tail vein. PET/CT scanning was performed 1 h post‐injection using a microPET/CT scanner (Inveon; Siemens, Germany). The acquired images were analyzed using the microQ Viewer software (Version 1.7.0.6; Siemens), with region of interest (ROI) delineating the tumor for quantitative analysis. Tumor FDG uptake was then calculated and expressed as the mean standardized uptake value (SUVmean) to facilitate statistical analysis of glucose uptake in vivo.

### Tumorigenicity Study

The animals were housed in specific pathogen free (SPF) condition. The number of animals was five per group. Once the tumor volume reached 2000 mm^3^, or the body weight decreased 15%, tumor‐bearing mice should be euthanized. A total of 5 × 10^6^ Ishikawa cells with shMGAT4A/EV, shC/EV, shMGAT4A/GAL9, and shC/GAL9 were subcutaneously inoculated into flanks of 4‐ to 6‐week‐old female athymic nude mice, respectively. The tumor size was measured every 3 days starting 2 weeks after implantation. The tumor volume was calculated with formula: tumor volume = 0.5 × length × width^2^.

### Luciferase Assay

Luciferase activity was assessed using a Luciferase Assay System (E1500, Promega, Madison, MI), following the manufacturer's protocol. Ishikawa cells in 24‐well plates were co‐transfected with psiCHECK‐2‐MGAT4A‐3'UTR (WT 3’UTR) or mutant MGAT4A‐3'UTR (Mutant 3’UTR) plasmids and pCMV‐RLuc, along with control miR‐NC, miR‐34a, miR‐449a, or miR‐449b. After 48 h, cells were lysed and luciferase activity was measured using a BioTek Synergy H1 microplate reader (Agilent, China). The results were expressed as the ratio of Firefly luciferase activities derived from the psiCHECK2 vector to Renilla luciferase activity.

### RNA Isolation, Quantitative Reverses Transcription‐PCR (qRT‐PCR), and RNA Interference

RNA isolation, qRT‐PCR, and RNAi were performed according to the manufacturers’ instructions. The mRNA levels were quantified by qRT‐PCR with SYBR Premix kit (DRR420A, TaKaRa, China) in ABI 7500 StepOne Plus Real‐Time PCR instrument (Applied Biosystem, Grand Island, NY). Expression levels of mRNA and miRNA expression were normalized to actin and U6 respectively, using the 2^−ΔΔCt^ method to calculate the relative expression levels. Sequences of the qPCR primers were listed in Table S5 (Supporting Information).

### Statistics Analysis

All statistical tests were justified for every figure. Each experiment was repeated at least three times. The mean with standard deviations (SDs) from at least triplicates was plotted. Survival analyses, including overall and disease‐free survival, were conducted using Kaplan–Meier curves and log‐rank tests. Pearson correlation was used to determine the correlations of variables. Differences in frequency distributions were analyzed using Fisher's exact test and χ^2^ test. Other differences between variables were analyzed using a two‐tailed Student's *t*‐test, one‐way ANOVA for comparisons involving more than two groups, and two‐way ANOVA for analyses with multiple variables. Statistical analyses were performed by GraphPad Prism 8.0 software. *p*‐value less than 0.05 was indicated as statistically significant.

## Conflict of Interest

The authors declare no conflict of interest.

## Author Contributions

Z.Z., J.S., and W.X. contributed equally to this work. J.Y, R.H., and Z.Z. conceptualized the work; Z.Z., J.S., W.X., and H.H. performed methodology; Z.Z., J.S., W.X., Q.Z., and H.F. performed formal analysis; Z.Z., J.S., W.X., Q.Z., H.F., L.Z., Y.H., X.H., N.S., X.R., and G.L. investigated the work; Z.Z., J.S., W.X., Q.Z., H.F., L.Z., Y.H., X.H., N.S., X.R., and G.L. performed data curation; J.Y., R.H., Z.Z., and J.S. wrote the original draft. J.Y., R.H., Z.Z., and J.S. wrote and edited the work; J.Y., R.H., H.H., and W.X. worked on resources, and J.Y., R.H., and H.H. supervised the work.

## Supporting information



Supporting Information

## Data Availability

The data that support the findings of this study are available from the corresponding author upon reasonable request.
