# Supplementary material for: MGAT4A/Galectin9‐Driven N‐Glycosylation Aberration as a Promoting Mechanism for Poor Prognosis of Endometrial Cancer with TP53 Mutation
Source: Adv Sci (Weinh). 2024 Nov 11;11(48):2409764. doi: 10.1002/advs.202409764 (PMC11672308; doi:10.1002/advs.202409764)
Supplement: Supplementary file 1 — Supporting Information [file ADVS-11-2409764-s001.docx]

Supporting Information

MGAT4A/Galectin9-driven *N-*glycosylation aberration as a promoting mechanism for poor prognosis of endometrial cancer with *TP53* mutation

Zhen Zhu, Jingya Sun, Weiqing Xu, Qinghe Zeng, Hanyi Feng, Lijuan Zang, Yinyan He, Xiao He, Na Sheng, Xuelian Ren, Guobin Liu, He Huang, Ruimin Huang*, and Jun Yan*

Z.Z., J.S. and W.X. contributed equally to this work.

* Corresponding authors.

This file includes:

**Figure S1.** Glycogenes dysregulation in TCGA-UCEC cohort (n=543) and our own cohort (n=60)

**Figure S2.** The associations of MGAT4A/MGAT4B expression with clinical features in EC patients

**Figure S3.** Galectin promoted ECs cells proliferation and invasion

**Figure S4.** MGAT4A and GAL9 co-promoted glucose metabolism in EC cells

**Figure S5.** p53 mutations drove tumor aggressiveness involving MGAT4A-mediated GLUT1 function and glucose metabolism

**Figure S6.** miR-34a and miR-449a/b as the negative regulators in EC

**Table S1.** The clinicopathological associations among 4 glycogene-clusters in TCGA-UCEC cohort

**Table S2.** The differentially-expressed glycogenes in Cluster A2 compared with other clusters (p<0.05, FDR<0.05)

**Table S3.** The associations between the clinicopathological parameters and MGAT4A protein level in 60 EC patients from our own cohort

**Table S4.** List of antibodies, recombinant protein, reagents and kits in this study

**Table S5.** List of oligonucleotide sequences

**Table S6.** List of oligonucleotide sequences for shRNA and siRNA


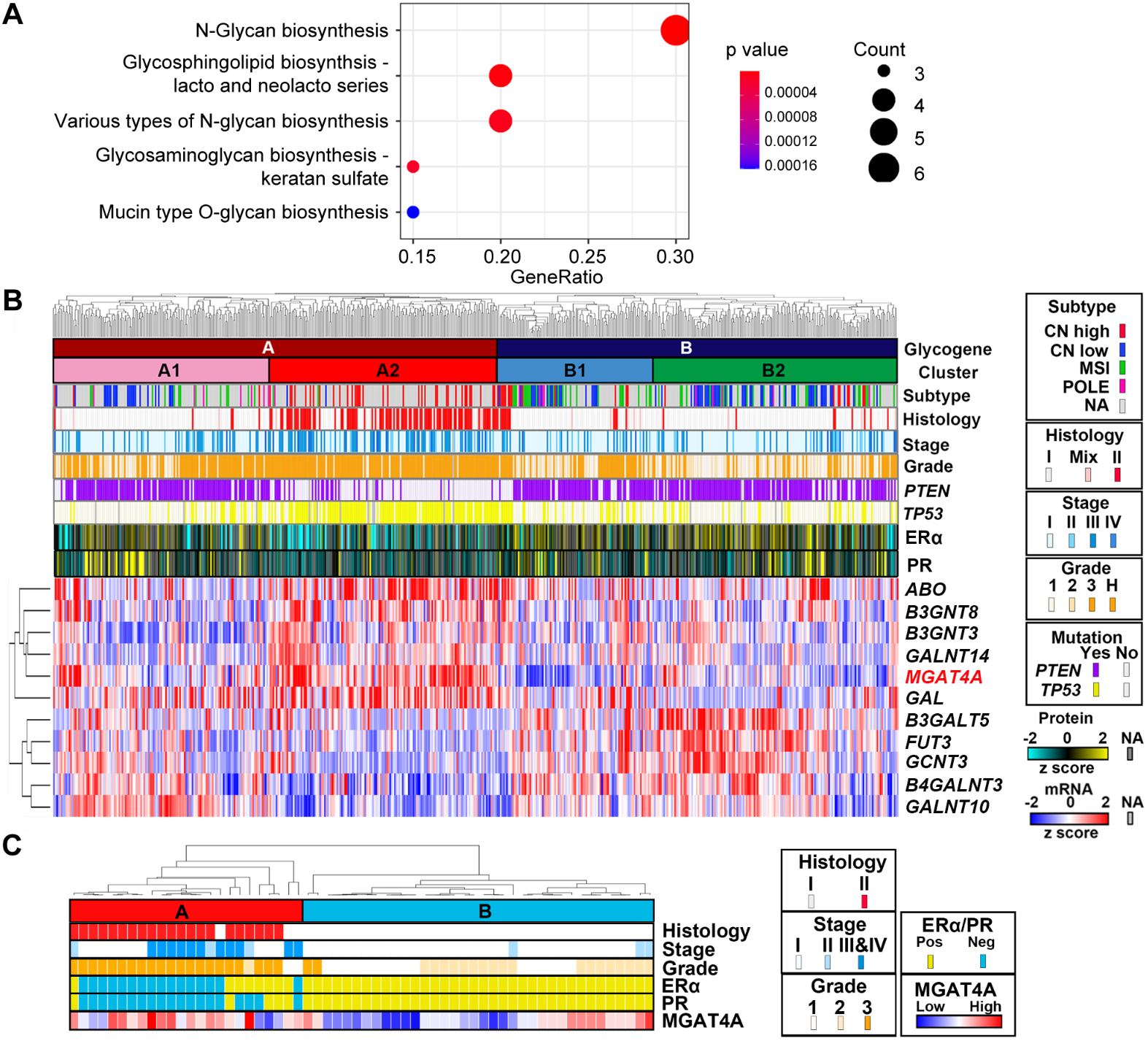


**Figure S1.** Glycogenes dysregulation in TCGA-UCEC cohort (n=543) and our own cohort (n=60). A) KEGG pathway analysis of different expressed genes in Cluster A2. The glycosylation related pathways were plotted. B) 11 glycogenes were identified to be significantly dysregulated in Cluster A2 comparing with clusters A1, B1 and B2 in TCGA-UCEC cohort. C) Clustering the EC patients from our own cohort using a combination of MGAT4A protein levels (by IHC scores) and the clinicopathological parameters (including histology, tumor stage, tumor grade, ERα status, and PR status).


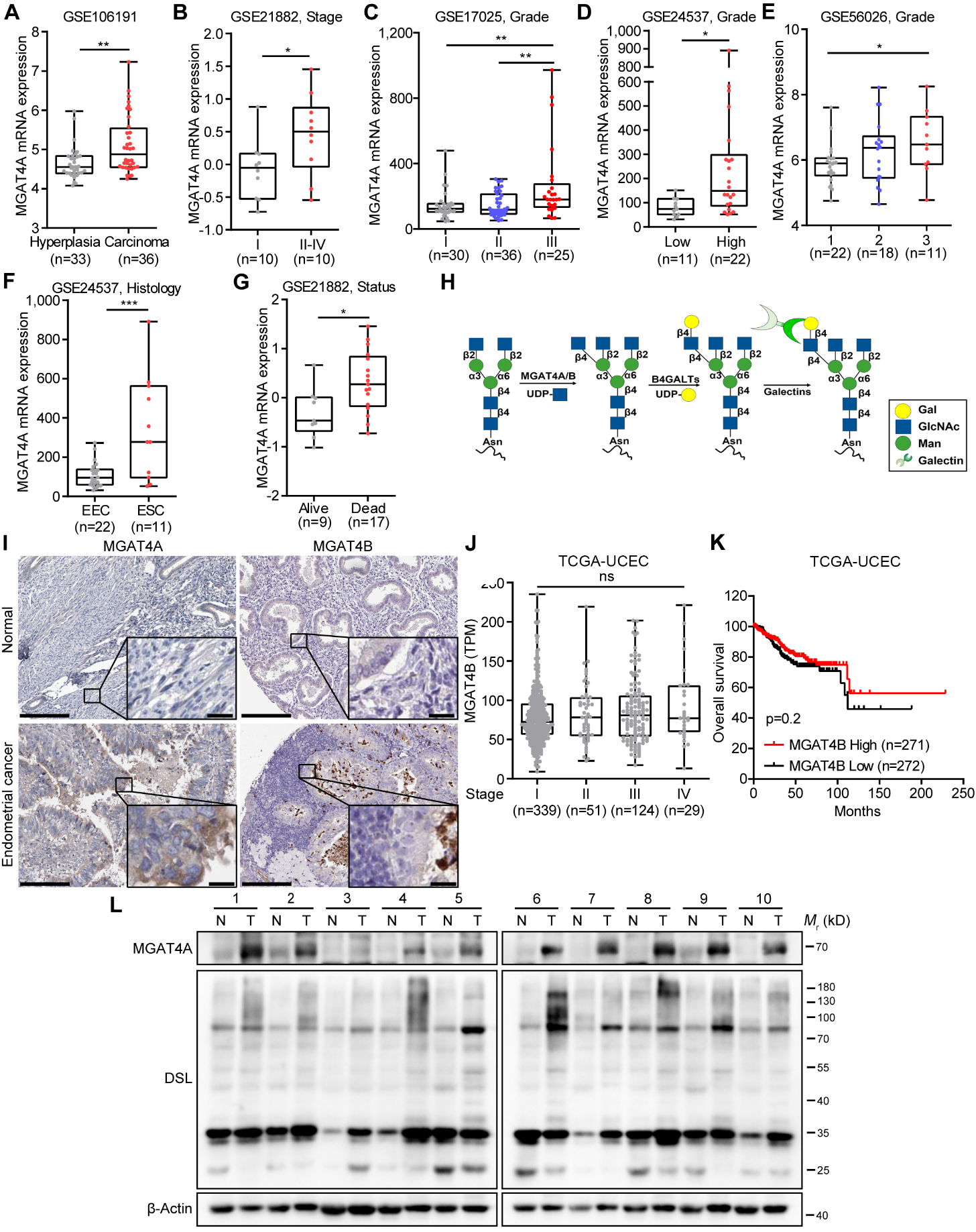


**Figure S2.** The associations of MGAT4A/MGAT4B expression with clinical features in EC patients. A) MGAT4A mRNA levels in hyperplasia and carcinoma endometrial tissues in GSE106191. B) The association of MGAT4A mRNA level with tumor stage (I *vs* II-IV) in GSE21882. C-E) The associations of MGAT4A mRNA level with tumor grade in GSE17025 (I-III, C), GSE24537 (Low *vs* High, D), and GSE56026 (1-3, E). F) The association of MGAT4A mRNA level with tumor histology in GSE24537 (EEC *vs* ESC). G) The association of MGAT4A mRNA level with survival status in GSE21882 (Alive *vs* Dead). H) Simplified branching of complex *N-*glycans involving MGAT4A/B proteins. GlcNAc branching of *N-*glycans is catalyzed by MGAT4A/B. LacNAc residues can be further recognized by galectins. I) From Human Protein Atlas endometrial cancer database, MGAT4A protein expression in endometrium normal tissue (#2021) and EC (#1851), and MGAT4B protein expression in endometrium normal tissue (#3364) and EC (#1058). Scale bar, 200 μm; inset, 20 μm. J) The association of MGAT4B mRNA level with tumor stage (I-IV) in EC patients from TCGA-UCEC cohort. K) Kaplan-Meier plot of the overall survival in EC patients from TCGA-UCEC dataset, stratified by MGAT4B mRNA level. L) The levels of MGAT4A protein and β-1,4-GlcNAc-branched *N*-glycan in paired EC and non-cancerous endometrial tissues (n=10), using Western blotting and DSL binding assays. N, non-cancerous endometrial tissue; T, EC tissue. p-values were calculated with unpaired two-tail Student’s *t*-test (A-B, D, F, and G) and one-way ANOVA (C, E, and J). *, p<0.05; **, p<0.01; ***, p<0.001; ns, p≥0.05.


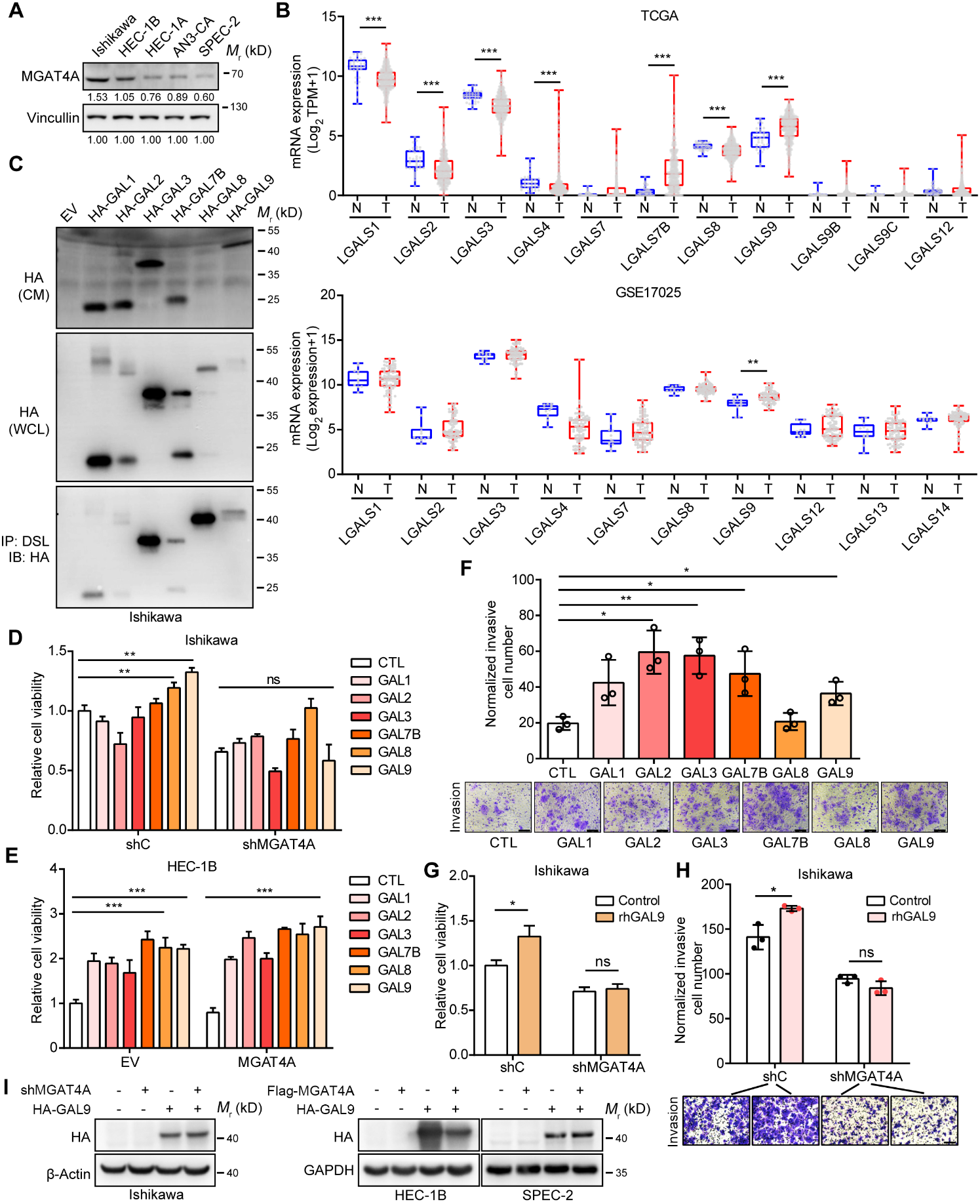


**Figure S3.** Galectin promoted ECs cells proliferation and invasion. A) The protein levels of MGAT4A in EC cell lines by Western blot analyses. B) The mRNA levels of galectin family members, including *LGALS1*, *2*, *3*, *4*, *7*, *8*, *9*, *12*, *13*, and *14*, in normal and EC tissue specimens from TCGA-UCEC and GSE17025 cohorts. C) Western blot analyses for protein levels of HA-tagged galectins in whole cell lysate (WCL) and conditional medium (CM) from Ishikawa cells transfected with different galectin family members (GAL1, 2, 3, 7B, 8, and 9). The interactions between these galectins and glycoproteins in WCL enriched by DSL-IP were examined in Ishikawa cells. D,E) The viability of Ishikawa (D) and HEC-1B (E) cells treated with CM from GAL1, 2, 3, 7B, 8, or 9-overexpressing Ishikawa cells by MTT assay. F) The invasion of Ishikawa cells treated with CM from GAL1, 2, 3, 7B, 8, or 9-overexpressing Ishikawa cells by transwell assay. G,H) The cell proliferation (G) and invasion (H) in Ishikawa shC and shMGAT4A cells treated with 2 μg mL^-1^ rhGAL9. I) GAL9 protein levels in the indicated Ishikawa, HEC-1B, and SPEC-2 cells after ectopic GAL9 overexpression. Scale bar, 100 μm. p-values were calculated with unpaired two-tail Student’s *t*-test (B), one-way ANOVA (F), and two-way ANOVA (D, E, G, and H). *, p<0.05; **, p<0.01; ***, p<0.001; ns, p≥0.05.


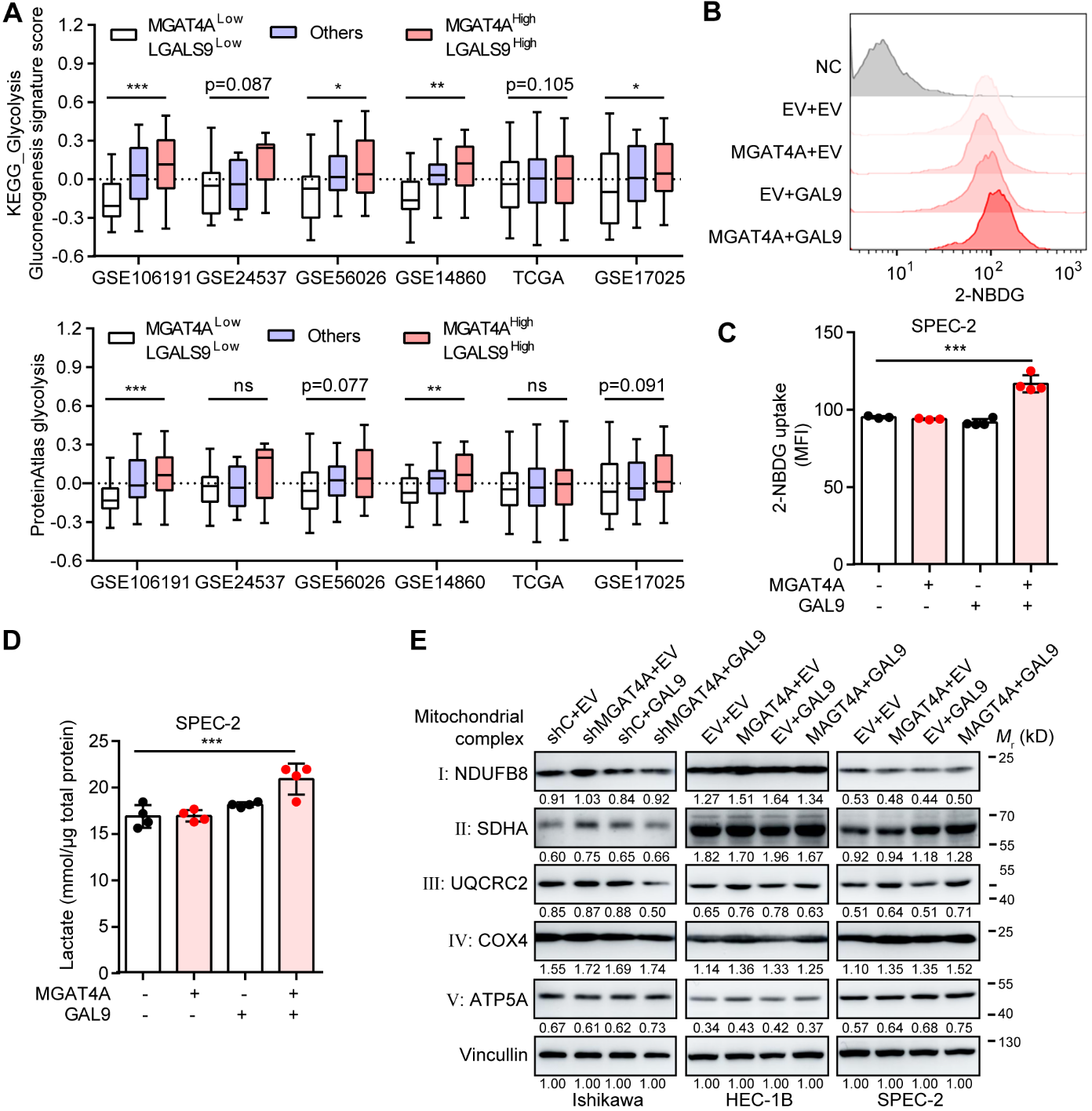


**Figure S4.** MGAT4A and GAL9 co-promoted glucose metabolism in EC cells. A) The associations between different glycolysis signature scores and MGAT4A/LGALS9 mRNA expression level in six public EC cohorts. B, C) 2-NBDG uptake in MGAT4A/GAL9 co-overexpressed SPEC-2 cells (B), quantified by MFI of 2-NBDG (C). D) Lactate production in MGAT4A/GAL9 co-overexpressing SPEC-2 cells after 48 h culture. E) Protein levels of mitochondrial complex components in Ishikawa, HEC-1B, and SPEC-2 cells with the knockdown/overexpression of MGAT4A and GAL9 by Western blot analyses. p-values were calculated with one-way ANOVA (A) and two-way ANOVA (C and D). *, p<0.05; **, p<0.01; ***, p<0.001; ns, p≥0.05.


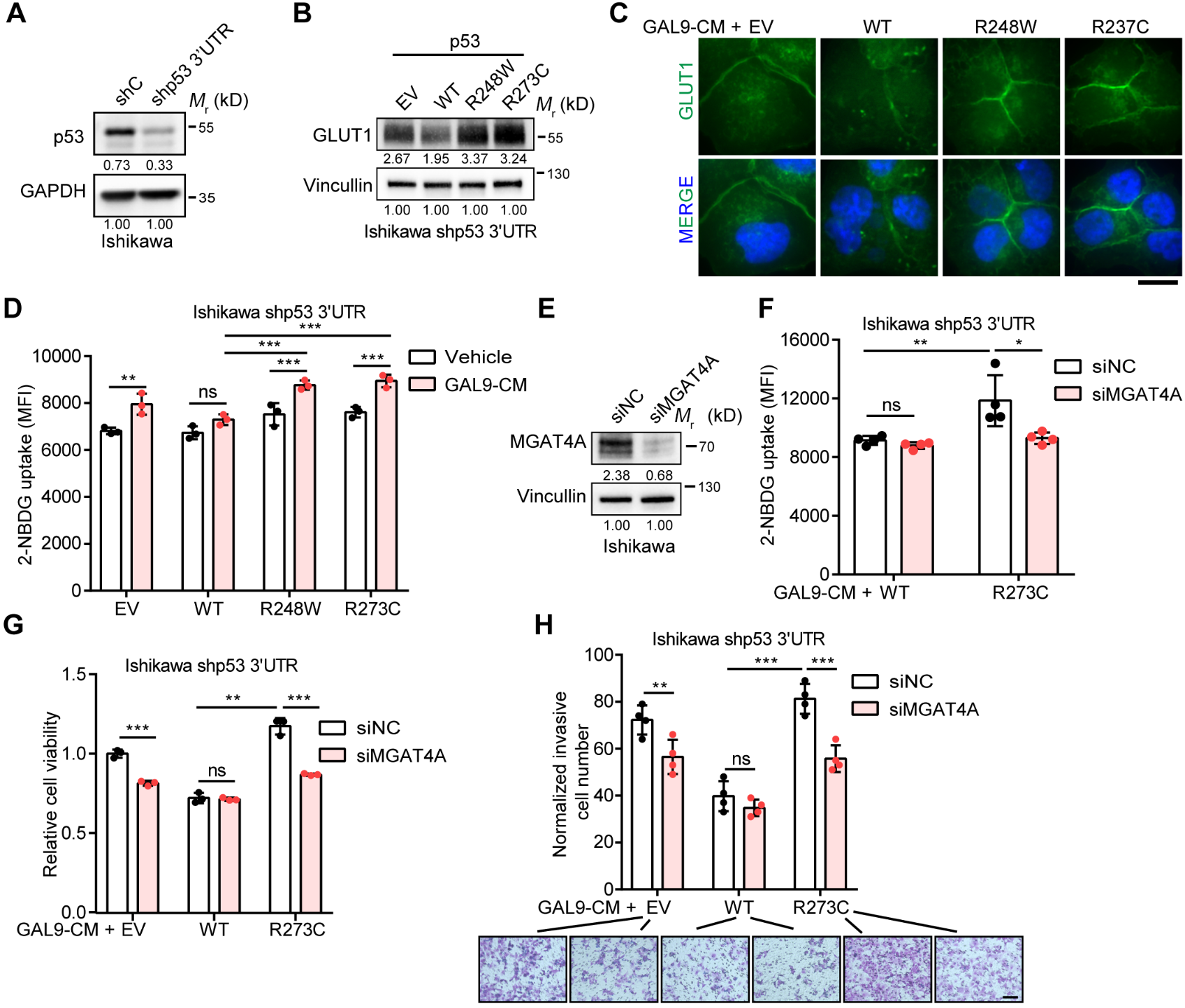


**Figure S5.** p53 mutations drove tumor aggressiveness involving MGAT4A-mediated GLUT1 function and glucose metabolism. A) p53 protein levels in Ishikawa cells with p53 stable knockdown using shRNA targeting its 3’UTR region (Ishikawa shp53 3’UTR). B) GLUT1 protein levels in Ishikawa shp53 3’UTR cells with stable ectopic expression of WT or mutated (R248W or R273C) p53. C) IF staining for GLUT1 (green) in Ishikawa shp53 3’UTR cells with WT or mutated p53, in conditional medium from GAL9-overexpressing Ishikawa cells (GAL9-CM). DAPI (blue) was used for nuclei staining. Scale bar, 20 μm. D) 2-NBDG uptake capability in Ishikawa shp53 3’UTR cells with WT or mutated p53, under GAL9-CM. E) MGAT4A protein levels in Ishikawa shp53 3’UTR cells with MGAT4A inhibition using siRNA targeting its mRNA (siMGAT4A). siNC, siRNA control. F) 2-NBDG uptake capability in Ishikawa shp53 3’UTR cells with WT or R273C p53 under GAL-CM, transfected with siMGAT4A/siNC. G) The relative viability of Ishikawa shp53 3’UTR cells with WT or R273C p53 under GAL-CM, transfected with siMGAT4A/siNC, using MTT assay. H) The normalized invasiveness of Ishikawa shp53 3’UTR cells with WT or R273C p53 under GAL-CM, transfected with siMGAT4A/siNC, using transwell assay. Scale bar, 100 μm. p-values were calculated with two-way ANOVA (D and F-H). *, p<0.05; **, p<0.01; ***, p<0.001; ns, p≥0.05.


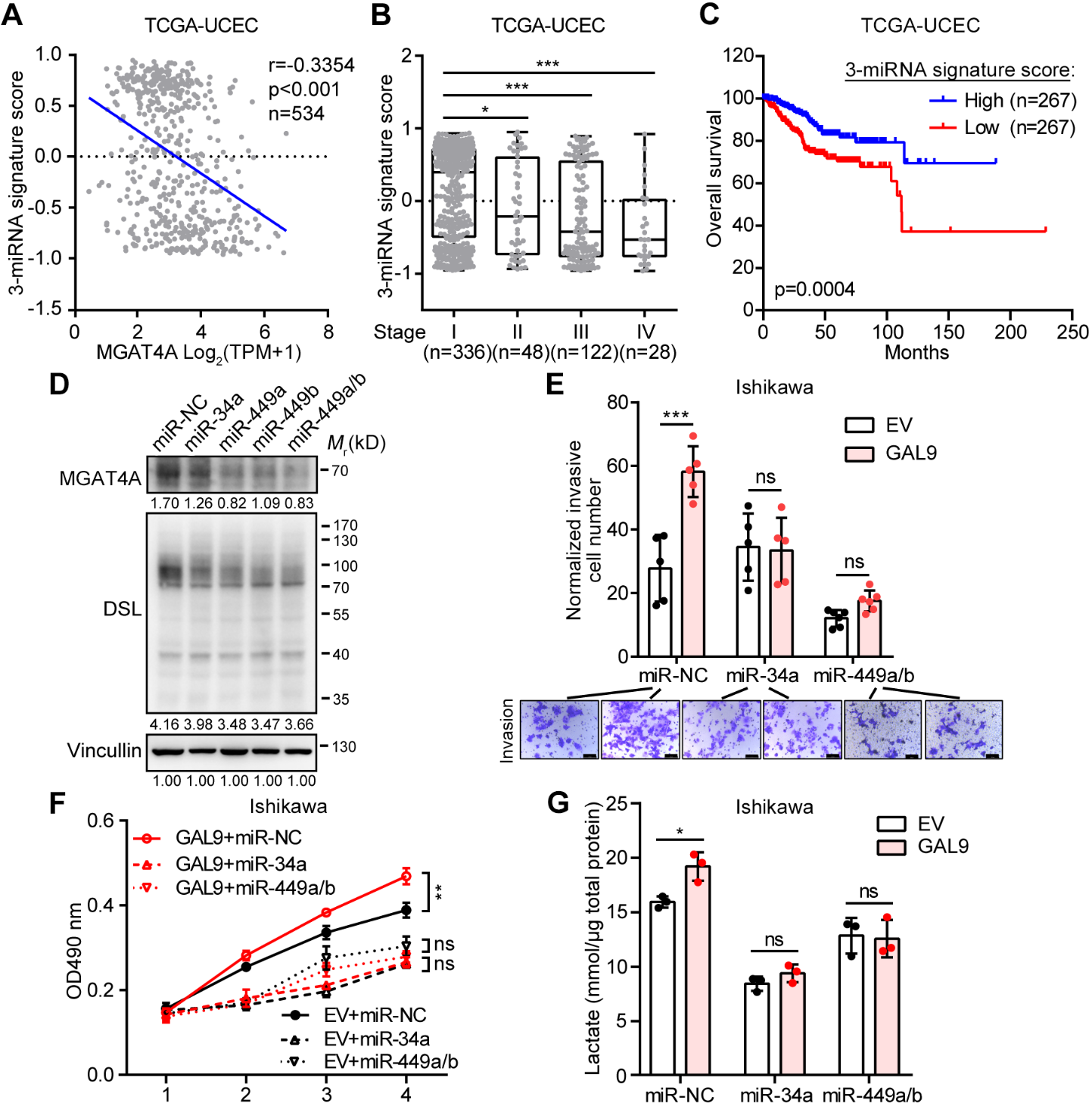


**Figure S6.** miR-34a and miR-449a/b as the negative regulators in EC. A-C) The associations of 3-miRNA signature score with MGAT4A expression (A), tumor stage (B), and overall survival (C) in EC patients from TCGA-UCEC cohort. D) The levels of MGAT4A protein and β1,4-GlcNAc modification mediated by miR-34a, 449a, and 449b in Ishikawa cells using Western blot analyses. E-G) Inhibitory effects of these three miRNAs on cell invasion (E), proliferation (F), and lactate production (G) in GAL9-overexpressing Ishikawa cells. Scale bar, 100 μm. p-values were calculated with one-way ANOVA (B) and two-way ANOVA (E-G). *, p<0.05; **, p<0.01; ***, p<0.001; ns, p≥0.05.

Table S1. The clinicopathological associations among 4 glycogene-clusters in TCGA-UCEC cohort.

|  | **Total** | **A1** | **A2** | **B1** | **B2** | **A1 B1 B2** | **A2 *vs* Others** |
| --- | --- | --- | --- | --- | --- | --- | --- |
|  | **(n=543)** | **(n=140)** | **(n=146)** | **(n=98)** | **(n=159)** | **(n=397)** | **p** |
| **Age (years)** | |  |  |  |  |  | **<0.0001** |
| **Mean** | 63.99 | 60.52 | 68.56 | 61.92 | 64.16 | 62.33 |  |
| **±SD** | ± 11.12 | ± 11.75 | ± 9.46 | ± 10.66 | ± 10.87 | ± 11.22 |  |
| **Histological** |  |  |  |  |  |  | **<0.0001** |
| **Endometrioid** | 407 | 131 | 47 | 82 | 147 | 360 |  |
| **Serous** | 114 | 6 | 89 | 11 | 8 | 25 |  |
| **Mix** | 22 | 3 | 10 | 5 | 4 | 12 |  |
| **MSI status** |  |  |  |  |  |  | **<0.0001** |
| **MSS** | 210 | 44 | 53 | 36 | 77 | 157 |  |
| **MSI-L** | 32 | 11 | 7 | 6 | 8 | 25 |  |
| **MSI-H** | 125 | 41 | 9 | 30 | 45 | 116 |  |
| **Indeterminant** | 3 | 0 | 1 | 1 | 1 | 2 |  |
| **NA** | 173 | 44 | 76 | 25 | 28 | 97 |  |
| **Grade** |  |  |  |  |  |  | **<0.0001** |
| **G1** | 98 | 18 | 2 | 18 | 60 | 96 |  |
| **G2** | 120 | 32 | 9 | 29 | 50 | 111 |  |
| **G3** | 314 | 90 | 128 | 48 | 48 | 186 |  |
| **High Grade** | 11 | 0 | 7 | 3 | 1 | 4 |  |
| **Stage** |  |  |  |  |  |  | **<0.0001** |
| **I** | 339 | 95 | 66 | 66 | 112 | 273 |  |
| **II** | 51 | 10 | 13 | 11 | 17 | 38 |  |
| **III** | 124 | 32 | 51 | 18 | 23 | 73 |  |
| **IV** | 29 | 3 | 16 | 3 | 7 | 13 |  |

Table S2. The differentially-expressed glycogenes in cluster A2 compared with other clusters (p<0.05, FDR<0.05).

| **Gene symbol** | **GENE ID** | **Description** | **Cluster** | **Cluster** |
| --- | --- | --- | --- | --- |
|  |  |  | **A2 *vs* others** | **A2 *vs* others** |
|  |  |  | **Log_2_ FC*** | **p** |
| GALNT14 | 79623 | polypeptide N-acetylgalactosaminyltransferase 14 | 1.631 | 5.26E-10 |
| GAL | 51083 | galanin and GMAP prepropeptide | 1.458 | 9.19E-11 |
| B3GNT3 | 10331 | UDP-GlcNAc:betaGal beta-1,3-N-acetylglucosaminyltransferase 3 | 1.406 | 1.58E-08 |
| MGAT4A | 11320 | alpha-1,3-mannosyl-glycoprotein 4-beta-N-acetylglucosaminyltransferase A | 1.340 | 8.85E-32 |
| ABO | 28 | ABO, alpha 1-3-N-acetylgalactosaminyltransferase and alpha 1-3-galactosyltransferase | 1.224 | 2.36E-08 |
| B3GNT8 | 374907 | UDP-GlcNAc:betaGal beta-1,3-N-acetylglucosaminyltransferase 8 | 1.009 | 1.22E-10 |
| B4GALNT3 | 283358 | beta-1,4-N-acetyl-galactosaminyltransferase 3 | -1.004 | 3.41E-14 |
| GALNT10 | 55568 | polypeptide N-acetylgalactosaminyltransferase 10 | -1.037 | 7.34E-22 |
| FUT3 | 2525 | fucosyltransferase 3 (Lewis blood group) | -1.354 | 5.47E-10 |
| B3GALT5 | 10317 | beta-1,3-galactosyltransferase 5 | -1.442 | 2.13E-08 |
| GCNT3 | 9245 | glucosaminyl (N-acetyl) transferase 3, mucin type | -1.583 | 6.00E-14 |

Notes: *FC, Fold change.

**Table S3.** The associations between the clinicopathological parameters and MGAT4A protein level in 60 EC patients from our own cohort

| **Characteristics** | **n** | **Expression of MGAT4A** | | **p** |
| --- | --- | --- | --- | --- |
|  |  | **high (n, %)** | **low (n, %)** |  |
| **Age (years)** |  |  |  | 0.0641 |
| < 60 | 36 | 14 (39%) | 22 (61%) |  |
| ≥ 60 | 24 | 16 (67%) | 8 (33%) |  |
| **T stage** |  |  |  | **0.0034** |
| T1 | 43 | 16 (37%) | 27 (63%) |  |
| T2-4 | 17 | 14 (82%) | 3 (18%) |  |
| **Tumor Grade** |  |  |  | 0.0910 |
| Low | 18 | 6 (33%) | 12 (67%) |  |
| High | 42 | 24 (57%) | 18 (43%) |  |
| **Ki67** |  |  |  | 0.4155 |
| < 20% | 6 | 4 (67%) | 2(33%) |  |
| ≥ 20% | 54 | 26 (48%) | 28 (52%) |  |
| **Estrogen receptor** |  |  |  | **0.0391** |
| **+** | 44 | 18 (41%) | 26 (59%) |  |
| **-** | 16 | 12 (75%) | 4 (25%) |  |
| **Progesterone receptor** |  |  |  | 0.0946 |
| **+** | 41 | 17(41%) | 24 (59%) |  |
| **-** | 19 | 13 (68%) | 6 (32%) |  |
| **Histology** |  |  |  | 0.2789 |
| Type I | 39 | 17 (44%) | 22 (56%) |  |
| Type II | 21 | 13 (62%) | 8 (38%) |  |
| **Menopausal status** |  |  |  | **0.0159** |
| pre/perimenopausal | 15 | 3 (20%) | 12 (80%) |  |
| postmenopausal | 43 | 25 (58%) | 18 (42%) |  |
| NA | 2 | 2 | 0 |  |
| **Myometrial invasion** |  |  |  | **0.0351** |
| < 50% | 39 | 16 (41%) | 23 (59%) |  |
| ≥ 50% | 20 | 14 (70%) | 6 (30%) |  |
| NA | 1 | 0 | 1 |  |

**Table S4.** List of antibodies, recombinant protein, reagents and kits in this study

| **Antibody** | **Company** | **Catalog #** | **RRID** | **Application (Dilution)** |
| --- | --- | --- | --- | --- |
| **1. Primary antibodies or lectin** | | | | |
| ATP5A | Proteintech | 14676-1-AP | AB_2061761 | WB (1:5K) |
| COX4 | Proteintech | 11242-1-AP | AB_2085278 | WB (1:5K) |
| Datura Stramonium Lectin (DSL) | Vector Laboratories | B-1185 | AB_2336384 | WB (1:1K), IF (1:100),  IP (1:100) |
| Flag-tag | Sigma-Aldrich | F1804 | AB_262044 | WB (1:1K), FACS (1:100) |
| Anti-Flag M2 affinity gel | Sigma-Aldrich | A2220 | AB_10063035 | IP (1:200) |
| Galectin-9 | Bioss | bs-0604R | AB_10857156 | IHC (1:100) |
| GAPDH | Santa Cruz | sc-32233 | AB_627679 | WB (1:10K) |
| GLUT1 | Abcam | ab652 | AB_305540 | WB (1:1K), IF (1:100),  IP (1:100), IHC (1:4K) |
| HA-tag | Santa Cruz | sc-7392 | AB_627809 | WB (1:1K), IF (1:100),  IP (1:100) |
| MGAT4A | Santa Cruz | sc-100785 | AB_2143042 | WB (1:1K), IHC (1:100) |
| NDUFB8 | Proteintech | 14794-1-AP | AB_2150970 | WB (1:5K) |
| p21 | Santa Cruz | sc-6246 | AB_628073 | WB (1:1K) |
| p53 | Santa Cruz | sc-126 | AB_628082 | WB (1:1K) |
| SDHA | Proteintech | 14865-1-AP | AB_11182164 | WB (1:5K) |
| UQCRC2 | Proteintech | 14742-1-AP | AB_2241442 | WB (1:5K) |
| Vinculin | Santa Cruz | sc-73264 | AB_1131292 | WB (1:10K) |
| **2. Secondary antibodies and other reagents** | | | | |
| Avidin agarose bead | Thermo Fisher | 20219 | NA | DSL coIP assay  (30 μL/reaction) |
| Normal mouse IgG | Santa Cruz | sc-2025 | AB_737182 | CoIP (1 mg) |
| Normal rabbit IgG | Santa Cruz | sc-2027 | AB_737197 | CoIP (1 mg) |
| Goat anti-Mouse IgG, HRP | Invitrogen | G-21040 | AB_2536527 | WB (1:10K) |
| Goat anti-Rabbit IgG, HRP | Invitrogen | G-21234 | AB_1500696 | WB (1:10K) |
| FITC-Avidin | BioLegend | 405101 | NA | IF (1:100) |
| Cy5 AffiniPure donkey anti-mouse IgG (H+L) | Jackson ImmunoResearch | 715-175-150 | AB_2340819 | IF (1:100) |
| Fluorescein (FITC)-AffiniPure goat anti-rabbit IgG (H+L) | Jackson ImmunoResearch | 111-095-144 | AB_2337978 | IF (1:100) |
| Protein A/G beads | Santa Cruz | sc-2003 | NA | IP |
| Streptavidin-HRP | Maxin | SP KIT-D1 | NA | WB (1:100) |
| **3. Recombinant protein** | | | | |
| rhGAL9 (recombinant human Galectin-9) | MedChemExpress | HY-P70535 | NA | 2 μg mL^-1^ |
| **Table S4.** List of antibodies, recombinant protein, reagents and kits in this study (*Cont’d*) | | | | |
| **Antibody** | **Company** | **Catalog #** | **RRID** | **Application (Dilution)** |
| **4. Reagents and kits** | | | | |
| BAY-876 | MedChemExpress | HY-100017 | NA | Ishikawa (2 μmol L^-1^) SPEC-2 (0.01 μmol L^-1^) |
| Blasticidin S | Sigma-Aldrich | SBR00022 | NA | Selection (1 μg mL^-1^) |
| 1×Carbo-Free blocking solution | Vector Laboratories | SP-5040 | NA | DSL assay |
| cOmplete mini, EDTA-free | Roche | 11836170001 | NA | Protein extraction |
| DSP, dithiobis(succinimidyl propionate) | Sangon Biotech | C110213 | NA | GLUT1 and galectins interaction (50 mmol L^-1^) |
| Galactose | Sigma-Aldrich | 59-23-4 | NA | Cell culture (10 mmol L^-1^) |
| Glucose | Sigma-Aldrich | 14431-43-7 | NA | Cell culture  (5 and 20 mmol L^-1^) |
| Glucose-free medium | Thermo Fisher | 11966 | NA | Tissue culture |
| Lactic acid assay kit | Nanjing Jiancheng Bioengineering Institute | A019-2-1 | NA | Cellular assay |
| Luciferase Assay System | Promega | E1500 | NA | Luciferase assay |
| Matrigel | Corning | 354234 | NA | *In vivo* tumorigenicity |
| 2-NBDG | Invitrogen | N13195 | NA | 10 μmol L^-1^ |
| PhosSTOP | Roche | 4906845001 | NA | Protein extraction |
| Puromycin | Sigma-Aldrich | P8833 | NA | Selection (1 μg mL^-1^) |
| SYBR Premix kit | TaKaRa | DRR420A | NA | qPCR assay |
| Transwell inserts (8 μm) pre-coated with Matrigel | Corning | 354165 | NA | Transwell assay |
| Tunicamycin | MedChemExpress | HY-A0098 | NA | Glycoprotein validation (10 μg mL^-1^) |

**Notes**: WB, Western blotting; IF, immunofluorescence; IP, immunoprecipitation.

Table S5. List of oligonucleotide sequences

| **Name** | **Direction** | **Nucleotide sequences (5’ - 3’)** |
| --- | --- | --- |
| **1. qPCR primer** | | |
| *RNU6* | RT-primer | CTCAACTGGTGTCGTGGAGTCGGCAATTCAGTTGAGAACGCTTC |
|  | qPCR-F | ACACTCCAGCTGGGACGCAAATTCGTGAAG |
| *hsa-miR-34a* | RT-primer | CTCAACTGGTGTCGTGGAGTCGGCAATTCAGTTGAGACAACCAG |
|  | qPCR-F | ACACTCCAGCTGGGTGGCAGTGTCTTAGCT |
| *hsa-miR-449a/b* | RT-primer | CTCAACTGGTGTCGTGGAGTCGGCAATTCAGTTGAGRCCAGCTA |
| *hsa-miR-449a* | qPCR-F | ACACTCCAGCTGGGTGGCAGTGTATTGTTA |
| *hsa-miR-449b* | qPCR-F | ACACTCCAGCTGGGAGGCAGTGTATTGTTA |
| Universal  reverse | qPCR-R | CTCAAGTGTCGTGGAGTCGGCAA |
| **2. Subcloning** | | |
| MGAT4A | F | ACGCTGGCCGGCCTGAATTCATGAGGCTCCGCAATGGAAC |
|  | R | GCAACCCCAACCCCCTCGAGTTCTCAGATGATCAGTTGGTGG |
| MGAT4A (3’UTR) | F | CCGCTCGAGTATGTGTCACTTGCTCCTAG |
|  | R | ATAAGAATGCGGCCGCCACAATCAACAGGACCAGTT |
| MGAT4A (3’UTR MUT) | F | TGTAAGATGCGTCATTGGTAGATTTGATACTTTTCATTTCAGT |
|  | R | CAAATCTACCAATGACGCATCTTACAGTACTGTCGAT |
| GLUT1 | F | GCTCTAGACCATGGAGCCCAGCAGCAAGA |
|  | R | CCGCTCGAGCGACTCACACTTGGGAATC |
| Flag-GLUT1 | F | ACAAAGACGATGACGACAAGGAGAGCATCCTGCCCACC |
|  | R | GTCGTCATCGTCTTTGTAGTCCCCATAGCGGTGGACCCA |
| GLUT1 (N45D) | F | ACAAAGACGATGACGACAAGCAGACATGGGTCCACCGCT |
|  | R | GTCGTCATCGTCTTTGTAGTCGTAGAACTCCTCGATCACCTT |
| TP53 (WT) | F | CTAGCTAGCATGGAGGAGCCGCAGTCAG |
|  | R | CCGCTCGAGTCAGTCTGAGTCAGGCCCTTC |
| TP53 (R248W) | F | CGGCATGAACTGGAGGCCCATCCTCACCATCATC |
|  | R | ATGGGCCTCCAGTTCATGCCGCCCATGCAGG |
| TP53 (R273C) | F | CTTTGAGGTGTGTGTTTGTGCCTGTCCTGGGAGAGA |
|  | R | GCACAAACACACACCTCAAAGCTGTTCCGTCCCAGTAG |
| LGALS1 | F | TACGCTGGCCGGCCTGAATTCATGGCTTGTGGT |
|  | R | GCAACCCCAACCCCCTCGAGTCAGTCAAAGGC |
| LGALS2 | F | GCGAATTCGAACTTGAGGTTAAGAACA |
|  | R | CCGCTCGAGTTATTCTTTTAACTTGAAA |
| LGALS3 | F | GTAGAATTCATGGCAGACAATTTTTCGCTCCA |
|  | R | CCGCTCGAGTTATATCATGGTATATGAAGCA |
| LGALS7B | F | TACGCTGGCCGGCCTGAATTCCCAGCCATGTCC |
|  | R | GCAACCCCAACCCCCTCGAGTCAGAAGATCCTCA |
| LGALS8 | F | TACGCTGGCCGGCCTGAATTCAGAATGATGTTGTC |
|  | R | GCAACCCCAACCCCCTCGAGCTACCAGCTCCT |
| LGALS9 | F | GTAGAATTCATGGCCTTCAGCGGTTCCC |
|  | R | CCGCTCGAGCTATGTCTGCACATGGGTC |

Table S6. List of oligonucleotide sequences for shRNA and siRNA

| **Name** | **Direction** | **Nucleotide sequences (5’ - 3’)** |
| --- | --- | --- |
| shC | F | CCGGGCGCGATAGCGCTAATAATTTCTCGAGAAATTATTAGCGCTATCGCGCTTTTTG |
|  | R | AATTCAAAAAGCGCGATAGCGCTAATAATTTCTCGAGAAATTATTAGCGCTATCGCGC |
| shMGAT4A-1 (puromycin) | F | CCGGCCGGATCTTACTCTGATTGTACTCGAGTACAATCAGAGTAAGATCCGGTTTTTG |
|  | R | AATTCAAAAACCGGATCTTACTCTGATTGTACTCGAGTACAATCAGAGTAAGATCCGG |
| shMGAT4A-2 (puromycin) | F | CCGGCCAGTCAATGTAGAAAGTTATCTCGAGATAACTTTCTACATTGACTGGTTTTTG |
|  | R | AATTCAAAAACCAGTCAATGTAGAAAGTTATCTCGAGATAACTTTCTACATTGACTGG |
| shTP53 3’UTR (puromycin) | F | CCGGGAGGGATGTTTGGGAGATGTACTCGAGTACATCTCCCAAACATCCCTCTTTTTG |
|  | R | AATTCAAAAAGAGGGATGTTTGGGAGATGTACTCGAGTACATCTCCCAAACATCCCTC |
| siNC | F | UUCUCCGAACGUGUCACGUUU |
|  | R | ACGUGACACGUUCGGAGAAUU |
| siMGAT4A | F | CAAAGAAACCAAAGACAAAUU |
|  | R | UUUGUCUUUGGUUUCUUUGUU |
